# Mechanisms of Dural Involvement in Cerebral Amyloid Angiopathy

**DOI:** 10.3390/cells15010026

**Published:** 2025-12-23

**Authors:** Marialuisa Zedde, Fabrizio Piazza, Rosario Pascarella

**Affiliations:** 1 Neurology Unit, Stroke Unit, Azienda Unità Sanitaria Locale-IRCCS di Reggio Emilia, Viale Risorgimento 80, 42123 Reggio Emilia, Italy; 2 SINdem Study Group “The Inflammatory Cerebral Amyloid Angiopathy and Alzheimer’s Disease Biomarkers”; 3 CAA and AD Translational Research and Biomarkers Laboratory, School of Medicine and Surgery, University of Milano-Bicocca, 20900 Monza, Italy; 4 Neuroradiology Unit, Ospedale Santa Maria della Misericordia, AULSS 5 Polesana, 45100 Rovigo, Italy

**Keywords:** cerebral amyloid angiopathy, CAA, sural, ICH, subdural, SDH, lymphatics, CAA-related inflammation

## Abstract

**Highlights:**

**What are the main findings?**

- This study identifies the potential involvement of the dura mater and its lymphatic system in the pathophysiology of Cerebral Amyloid Angiopathy (CAA), suggesting that these structures may play a role in the clearance of amyloid-beta (Aβ) and maintenance of cerebrospinal fluid homeostasis.
- The research highlights a significant association between CAA and subdural hematoma (SDH), indicating that patients with CAA may face an increased risk of hemorrhagic complications, which complicates their clinical management.

**What are the implications of the main findings?**

- Recognizing dural involvement in CAA emphasizes the need for further investigation into the role of meningeal lymphatics in amyloid clearance and cerebrovascular health, potentially leading to novel therapeutic approaches.
- Understanding the connection between CAA and SDH may guide clinicians in monitoring and treating patients more effectively, particularly in identifying those at higher risk for hemorrhagic events and adjusting management strategies accordingly.

**Abstract:**

Cerebral Amyloid Angiopathy (CAA) is a neurovascular condition characterized by the accumulation of amyloid-beta (Aβ) in the walls of small blood vessels, particularly affecting the leptomeninges and cortical regions in elderly populations. Initially recognized for its association with spontaneous lobar intracerebral hemorrhage, recent studies have highlighted the broader implications of CAA on cognitive decline and vascular health. This narrative review aims to elucidate the mechanisms of dural involvement in CAA, an aspect that has been largely overlooked in existing literature. This paper provides a detailed examination of the potential role of the dura mater and its associated lymphatic system in the clearance of interstitial amyloid and the maintenance of cerebrospinal fluid (CSF) homeostasis. Dural lymphatic vessels may facilitate the efflux of Aβ from the brain, and any impairment in this drainage system could contribute to the pathological accumulation of amyloid, exacerbating CAA and its neurological consequences. Additionally, the significant association between CAA and subdural hematoma (SDH) has been explored, indicating that the presence of SDH may complicate the clinical management of CAA patients by signaling an increased risk of hemorrhagic events. The mechanisms linking CAA and SDH, including vascular fragility and chronic inflammatory processes, are discussed to provide insight into potential pathways for therapeutic intervention.

## 1. Introduction

Cerebral amyloid angiopathy (CAA) is defined by the deposition of amyloid β (Aβ) in the walls of small arteries, arterioles, and capillaries located within the leptomeninges, cerebral cortex, and cerebellar cortex. This condition is commonly found in older adults, with moderate-to-severe cases detected in approximately 25% of autopsied brains from individuals averaging 84.9 years of age [1]. Initially identified as a major contributor to spontaneous lobar intracerebral hemorrhage, CAA has been associated with this form of hemorrhage across various hospital cohorts [1,2]. CAA-related lobar hemorrhages have high morbidity and mortality rates [3], presenting an annual recurrence rate of around 7.4% [4], which surpasses that of most other stroke types. Additionally, these hemorrhages may lead to subarachnoid bleeding over brain convexities, potentially resulting in transient focal neurological episodes [5]. CAA is also independently linked to cognitive decline, with autopsy findings of moderate-to-severe CAA correlating with accelerated cognitive decline in late life among community-dwelling individuals, even when considering Alzheimer’s disease and other neurodegenerative disorders [6]. The exact mechanisms connecting CAA to cognitive impairment remain unclear but likely involve non-hemorrhagic brain injuries such as microinfarcts [7] and white matter changes [8,9,10]. Understanding the progression from initial cerebrovascular amyloid deposition to both hemorrhagic and non-hemorrhagic brain injuries is essential for developing therapies aimed at modifying disease outcomes. However, existing neuropathological studies predominantly rely on cross-sectional designs, limiting their capacity to clarify timing and causation. A promising strategy to enhance these studies involves monitoring mechanistic processes through biomarkers in CAA patients. Diagnosis can be informed by characteristic hemorrhagic lesions using clinical–radiological criteria, including the MRI-based Boston [11] or CT-based Edinburgh [12,13] criteria. Genetic diagnosis is also applicable for hereditary forms, such as Dutch-type CAA [14,15]. Research has revealed aspects of CAA, like an impaired cerebrovascular response to visual stimuli, which may not be detectable at autopsy [16,17]. Longitudinal studies of hereditary mutation carriers can further elucidate disease progression before the emergence of brain lesions and symptoms. Our objective is to establish a pathophysiological framework and timeline for CAA progression, detailing early vascular changes leading to symptomatic non-hemorrhagic and hemorrhagic injuries, while specifically excluding rare non-Aβ forms of cerebrovascular amyloid angiopathy. Our approach is data-driven, incorporating neuropathological analyses, in vivo biomarker studies of both sporadic and hereditary CAA, animal model research, and findings from recent studies concerning early-onset iatrogenic CAA following presumed childhood exposure to Aβ [18]. Each source of data presents limitations, such as discrepancies between animal models and human disease, variations in clinical trajectories between hereditary and sporadic CAA [14], and imperfect correlations between biomarker readings and the underlying pathophysiology. Nevertheless, this proposed framework sets the stage for future biomarker and intervention trials for CAA, deepens our understanding of amyloid dynamics in Alzheimer’s disease (AD) [19], and addresses non-amyloid-related small vessel diseases [20]. CAA is noted for the accumulation of beta-amyloid in small vessel openings, specifically arterioles, capillaries, and leptomeningeal and cortical venules. Within this framework, the potential involvement of dural arterioles is not explicitly addressed, possibly due to the differing vascularization and embryological origins of the dura mater, primarily supplied by the dural branches of the external carotid artery and the vertebral artery. Another limitation is the insufficient examination and description of the dura mater in both animal and human biopsy specimens, where observations tend to focus on leptomeningeal and cortical small vessels rather than pachymeningeal arterioles [21]. Despite this, spontaneous subdural hematomas have gained significant attention as a hemorrhagic manifestation of CAA, although there is no consensus on the cause–effect relationship between the two. Furthermore, cadaveric dura mater has been identified as a potential disease vector in iatrogenic forms of CAA. These factors highlight the possibility, albeit not prominently featured, of dural involvement in CAA. This narrative review aims to explore the biological mechanisms that may underlie dural involvement in CAA and its associated clinical manifestations.

## 2. CAA Pathophysiology Model and Dural Issues

Research has identified a sequential progression of CAA that consists of four primary stages [22]: (1) cerebrovascular amyloid deposition, (2) alterations in cerebrovascular physiology, (3) non-hemorrhagic brain injury, and (4) hemorrhagic brain lesions. Each of these stages is supported by in vivo biomarker data from Dutch-type CAA mutation carriers, as well as biomarker information from sporadic or iatrogenic CAA, histopathological analyses, and findings from transgenic mouse models [23,24].

The first stage involves the earliest detectable event in CAA, which is vascular amyloid deposition, particularly observed in Dutch-type mutation carriers. The specific triggers for this deposition remain unclear, but factors such as the characteristics of Aβ, age, and APOE genotype are likely contributors [19]. Notably, there is no significant overproduction of Aβ in either sporadic or Dutch-type CAA [25], indicating that the observed deposition may result from either increased aggregation or decreased clearance mechanisms. Deposition of cerebrovascular amyloid can be inferred from reduced concentrations of Aβ in cerebrospinal fluid (CSF) [26], which demonstrate an inverse correlation with neuritic plaques (largely absent in Dutch-type CAA). Consistently low levels of Aβ42 and Aβ40 have been noted in sporadic CAA [23] as well as in both presymptomatic and symptomatic Dutch-type CAA. These decreases can be detected in mutation carriers as early as their mid-20s, approximately 30 years prior to the onset of symptomatic intracerebral hemorrhage. Additionally, reductions in plasma Aβ levels have been observed in older presymptomatic carriers. The pattern of Aβ reduction in Dutch-type CAA contrasts with that seen in sporadic Alzheimer’s disease (AD), which typically shows decreased Aβ42 without a significant reduction in Aβ40 [23,27]. Furthermore, the absolute concentrations of Aβ in Dutch-type CAA appear lower than the ones found in autosomal dominant Alzheimer’s disease at both presymptomatic and symptomatic stages. Amyloid PET imaging with Pittsburgh compound B has demonstrated amyloid deposition in presymptomatic carriers, although changes manifest later than reductions in CSF Aβ levels. Increased retention of Pittsburgh compound B in presymptomatic and symptomatic carriers correlates with lower concentrations of CSF Aβ40 (r = −0.55), indicating vascular amyloid deposition. However, Pittsburgh compound B imaging is less sensitive for detecting Dutch-type CAA compared to the pathology of autosomal dominant Alzheimer’s disease, as carriers of autosomal dominant mutations exhibit significantly greater retention at lower degrees of CSF Aβ reduction. Evidence from early-onset CAA, induced by iatrogenic exposure to exogenous Aβ, sheds light on the timeline from initial amyloid deposition to symptomatic hemorrhage, which has been documented in humans, typically resulting in hemorrhage during the third to fifth decades of life [18,28,29]. Although these cases are rare, they raise important mechanistic questions regarding the behavior of amyloid and the factors influencing vascular deposition. While histopathological samples from presymptomatic Dutch-type CAA carriers are scarce, neuropathological observations suggest that Aβ is initially deposited in the outer basement membranes surrounding smooth muscle cells [30]. Some cases show substantial Aβ deposition in the capillary basement membranes, which may align more closely with AD pathology than with arteriolar CAA. The anatomical features of early CAA can be modeled through in vivo imaging of transgenic mouse models, such as APPswe/PS1dE9 and Tg2576 mice, which display a gradual accumulation of vascular Aβ beginning in larger arterioles [31,32]. Kinetic modeling indicates that amyloid accumulation is primarily driven by the propagation of existing deposits rather than the initiation of new ones [32]. Moreover, the presence of the APOE ε4 allele may further enhance vascular Aβ accumulation [33].

In stage two, notable alterations in vascular physiology become a significant concern. Impaired cerebrovascular reactivity has been recognized as a hallmark of sporadic CAA, with studies employing transcranial Doppler and BOLD fMRI techniques demonstrating reduced response amplitudes and delayed peak responses during visual stimulation [16,34,35]. Remarkably, these impairments are evident even in presymptomatic carriers of the Dutch-type CAA mutation, suggesting that issues with vascular reactivity can develop long before any structural brain damage is detectable via MRI [17]. Research indicates that differences in BOLD fMRI responses between mutation carriers and non-carriers begin to emerge around the ages of 34 to 38, roughly twenty years prior to the onset of symptomatic intracerebral hemorrhage. Longitudinal studies have shown a progressive decline in vascular reactivity over time, particularly among presymptomatic carriers, highlighting the early and evolving nature of these changes [35,36]. Histopathological evidence from mouse models indicates that the loss of smooth muscle cells in arterioles is associated with the impaired vascular physiology observed. For example, in Tg2576 transgenic mice, significant amyloid-beta (Aβ) deposition at 19 months was linked to compromised vascular reactivity, while younger mice without Aβ deposition maintained normal vascular function. Additional studies involving APPswe/PS1dE9 transgenic mice have supported these findings, revealing a similar impairment in reactivity that appears to correlate more closely with the loss of vascular smooth muscle cells than with the overall Aβ burden [37,38]. The implications of these vascular changes are substantial, as they may contribute not only to non-hemorrhagic brain injuries but also to reduced interstitial fluid clearance, which is crucial for preserving brain health. Experimental studies suggest that these impairments hinder the clearance of fluorescent tracers from the brain, indicating a direct connection between vascular reactivity and fluid clearance [39,40,41]. Mathematical models further bolster this idea, proposing that smooth muscle cell contractions are vital for efficient interstitial fluid clearance [42,43]. Ultimately, if the vascular alterations associated with CAA obstruct the clearance of soluble extracellular Aβ, this could establish a self-reinforcing cycle that promotes additional Aβ deposition within blood vessels and the brain parenchyma, thereby contributing to the formation of senile plaques characteristic of AD [19]. The intricate relationship between vascular health and amyloid pathology emphasizes the complexity of CAA and its far-reaching implications for neurological health.

In the advanced stages of CAA, significant non-hemorrhagic injuries to brain tissue manifest, characterized by distinct focal damage and microstructural alterations that differ from typical hemorrhagic lesions. Various non-hemorrhagic MRI findings are linked to sporadic CAA, including lobar lacunes, microinfarcts, white matter hyperintensities, and visible perivascular spaces in the centrum semiovale [7,8,9,10,44,45]. Moreover, diffusion tensor imaging (DTI) has uncovered ultrastructural markers such as increased mean diffusivity and decreased histogram fractional anisotropy, which are associated with cognitive decline, particularly in executive function and processing speed. Research on patients with Dutch-type CAA has revealed that these non-hemorrhagic brain injuries likely commence around 10 to 15 years before the average age of symptomatic hemorrhage, escalating rapidly over time [46,47]. This timeline suggests that vascular dysfunction occurs prior to the onset of non-hemorrhagic injuries, aligning with findings indicating that changes in BOLD fMRI responses mediate the relationship between amyloid PET signals and white matter hyperintensity volumes [45]. However, it remains uncertain whether the observed white matter changes impact cognition, as cognitive impairment has not been detected in presymptomatic carriers before their first intracerebral hemorrhage, with only 12 carriers evaluated at a mean age of 34 [47]. The neuropathological basis for DTI abnormalities in sporadic CAA has been examined through correlations with histopathological data. Notably, loss of fractional anisotropy in specific white matter tracts, such as the inferior longitudinal fasciculus and anterior thalamic radiation, has been associated with tissue rarefaction and decreased axonal density, while increased mean diffusivity corresponds to reduced myelin density [48]. Microinfarcts in these areas exhibit similar patterns of altered DTI parameters. These microinfarcts, which preferentially occur in vascular border zones, may arise from hypoperfusion. Ex vivo analyses of brains from patients with sporadic CAA indicate that these lesions are frequently found in regions of severe vascular amyloid pathology, characterized by thickened vessel walls and significant Aβ deposition, leading to a loss of vascular smooth muscle cells and diminished reactivity [49,50]. Another notable non-hemorrhagic MRI marker associated with both sporadic and hereditary CAA is the count of visible perivascular spaces in the centrum semiovale (CSO-PVS). A high CSO-PVS count, defined as more than 20 visible lesions per axial MRI slice, is considered relatively specific for advanced CAA and has been included in the latest Boston diagnostic criteria for probable CAA [11]. Increased CSO-PVS counts have been documented in symptomatic patients but not in presymptomatic carriers [47], implying that such enlargement occurs later in the disease progression. Histopathological investigations indicate that most enlarged CSO-PVS are located around penetrating arterioles in the white matter, which originate from cortical vessels exhibiting extensive Aβ deposition and loss of smooth muscle cells [11]. Furthermore, a correlation has been established between CSO-PVS counts and amyloid burden as measured by Pittsburgh compound B PET scans [51,52]. This proximity raises the intriguing hypothesis that the enlargement of perivascular spaces may result directly from impaired clearance of interstitial fluid, potentially exacerbating the pathological processes associated with CAA.

Stage four of CAA is characterized by significant hemorrhagic brain lesions, primarily manifesting as intracerebral hemorrhages known as macrobleeds. These macrobleeds are typically larger than 1 cm and are predominantly located in the lobar regions and superficial cerebellar cortex [53,54]. In addition to macrobleeds, T2-weighted MRI has identified other hemorrhagic manifestations associated with both sporadic and hereditary forms of CAA, including cerebral microbleeds, cortical superficial siderosis, and convexity subarachnoid hemorrhage. Cerebral microbleeds are small, round lesions that appear in similar brain regions as macrobleeds [55]. Cortical superficial siderosis represents a more chronic condition, characterized by the presence of blood products in the cerebral and cerebellar sulci, likely resulting from previous subarachnoid hemorrhages [56,57]. The interplay among these various types of hemorrhagic lesions is complex. Individual patients with CAA may exhibit a predisposition to specific hemorrhagic subtypes [53]. Research indicates that all these hemorrhagic lesions tend to occur concurrently during a distinct phase of the disease, following the initial vascular amyloid deposition and preceding non-hemorrhagic brain injury. In Dutch-type CAA, both microbleeds and cortical superficial siderosis first appear around the same age as the initial intracerebral hemorrhage, typically at a mean age of 54 years, occurring at least a decade after the first signs of non-hemorrhagic changes [46].

Despite the high prevalence of moderate-to-severe CAA pathology identified in older autopsy studies, which report a pooled estimate of 23.0% in the general population (mean age 80–85 years), the occurrences of lobar microbleeds (7.1%) and cortical superficial siderosis (0.8%) remain relatively low [1]. This discrepancy suggests that hemorrhagic events are a later development in the progression of CAA.

The underlying vascular pathology reveals a notable delay between the deposition of amyloid (detectable in mice at 12 months of age) and the emergence of microbleeds (detectable in mice at 16–20 months) [58,59]. Histopathological studies show that patients with a higher count of microbleeds (>80) often exhibit worse overall severity of CAA compared to those with lower counts (<80) [50]. Interestingly, the severity of CAA appears reduced near microbleeds, suggesting that the vascular remodeling associated with these lesions—characterized by smooth muscle cell loss and fibrin replacement—could be a contributing factor to the bleeding rather than a consequence of it.

Additionally, the presence of increased reactive astrocyte and activated microglia staining surrounding remodeled vessels indicates that inflammation may play a significant role in the vascular changes linked to hemorrhage [60]. It is also important to note that vascular remodeling is not exclusive to CAA, as severe arteriolosclerosis exhibits similar pathological features [61]. Identifying vessels involved in cortical superficial siderosis is more complex due to the diffuse nature of leptomeningeal hemorrhage; however, histological analysis reveals the presence of iron-positive hemosiderin in the subarachnoid space and underlying cortex, in conjunction with evidence of increased severity of CAA in leptomeningeal vessels [62,63].

What stands out in this model is the absence of mention regarding possible dural involvement, which appears to be understudied and perhaps neglected, similar to venular involvement [64]. The potential for dural involvement in CAA hinges on hypothetical mechanisms involving the dura mater and the associated dural lymphatics.

## 3. Involvement of the Dural Lymphatics

The noted decline in Aβ clearance in aging individuals and those with AD suggests that a reduction in the body’s natural mechanisms for Aβ clearance may contribute to its accumulation in the human brain, affecting not only AD but also cerebral amyloid angiopathy (CAA) [65,66]. Aβ removal from the brain’s interstitial space occurs through several processes, including local cellular degradation, receptor-mediated transport across the blood–brain barrier (BBB), and perivascular exchange into the cerebrospinal fluid (CSF) compartment via the glymphatic system [67,68,69,70].

The identification of a meningeal lymphatic vascular system in mice has profound implications for our understanding of interstitial homeostasis in the brain and central nervous system (CNS), CSF physiology, and immune surveillance within the CNS [67,68,71,72]. These findings have recently been supported in three human subjects and nonhuman primates by Absinta et al. [73,74], who employed contrast-enhanced magnetic resonance imaging along with immunohistochemical techniques to visualize meningeal lymphatic vessels marked by specific lymphatic endothelial cell markers. In both mice and humans, meningeal lymphatic vessels are located alongside large blood vessels and cranial nerves within the dura mater, reflecting distribution patterns seen in the peripheral lymphatic system. In mice, these vessels facilitate the absorption of macromolecules from the brain and CSF, transporting these solutes to the deep cervical lymph nodes (DCLNs). This efflux pathway provides a structural basis for the experimental observation that substances introduced into the CNS tend to accumulate in the DCLNs [74,75,76]. While the function of meningeal lymphatics remains largely unexplored, it is hypothesized that this pathway plays a vital role in clearing pathological waste products, such as Aβ, from the brain.

An evaluation of the structure and function of meningeal lymphatic vessels in the context of AD has been suggested [77]. It remains unclear whether Aβ is deposited in the walls of meningeal lymphatic vessels, similar to the deposits observed along leptomeningeal and intraparenchymal arteries in cases of CAA, which affects over 90% of AD brains [78]. Postmortem human dural tissue samples containing the superior sagittal sinus were collected and processed. The Braak neurofibrillary tangle and CERAD amyloid plaque scores associated with AD pathologies were evaluated [79,80]. Coronal sections (7 μm) of paraffin-embedded human dura and superior sagittal sinus were prepared and subjected to immunoreactivity analysis. Whole-slide fluorescence microscopy was utilized to visualize podoplanin (PDPN), a marker for lymphatic endothelial cells, across the extensive histological samples of the superior sagittal sinus (SSS). Individual PDPN+ lymphatic vessels were identified using high-resolution microscopy with spectral unmixing to eliminate tissue autofluorescence. PDPN+ vessels were found in 19 out of 21 subject samples, including all 6 AD subjects, 4 out of 5 control subjects, and 9 out of 10 subjects with mixed dementia or other neurological conditions. Two distinct morphologies of PDPN+ lymphatic vessels were noted in human postmortem SSS samples. One type exhibited typical initial lymphatic characteristics, featuring a single layer of endothelium, an absence of smooth muscle or red blood cells, an unobstructed lumen, and an irregular shape (“Type 1” lymphatic vessels). The second type (“Type 2”) showed an irregular endothelial border and contained material within the lumen, resembling the lymphatic vessels described in human autopsy samples by Louveau et al. [81]. Unlike meningeal arteries and veins, both vessel types were negative for the blood endothelial cell marker CD31, confirming that PDPN+ Type 1 and Type 2 vessels are not meningeal blood vessels. The distribution of Type 1 and Type 2 lymphatic vessels within dural tissue varied, with Type 1 vessels found in the periosteal and meningeal layers of the dura mater, while Type 2 vessels were located between the SSS and the periosteal layer of the dura. Consistent with findings in rodent studies, lymphatic vessels of both types were negative for the smooth muscle cell marker smooth muscle actin.

Both Type 1 and Type 2 lymphatic vessels were easily identifiable in control and AD subjects. No significant differences in vessel circumference were observed between AD and control groups, with average measurements of 354 ± 55 μm and 381 ± 76 μm, respectively. The presence of Aβ in the walls of leptomeningeal and intraparenchymal cerebral arteries is commonly seen in AD patients and is thought to reflect the role of perivascular spaces as pathways for Aβ efflux from the brain parenchyma [82]. If the meningeal lymphatic system participates in Aβ clearance, Aβ deposition may also be observed along the meningeal lymphatic vessels in AD subjects. To investigate this hypothesis, sequential sections of the cortex and subarachnoid space (SSS) were labeled with the 6E10 and 4G8 Aβ antibody clones, which have differential detection capabilities for prefibrillar oligomeric Aβ. Specifically, the 4G8 antibody binds to both fibrillar and prefibrillar oligomeric Aβ, whereas the 6E10 antibody exclusively binds to fibrillar Aβ [83]. As expected, Aβ immunoreactivity was largely absent in frontal cortical sections from control subjects, while AD subjects exhibited dense Aβ immunoreactivity in frontal cortical regions. In the dura mater, Aβ reactivity was clone-specific in both AD and control subjects, with widespread diffuse immunoreactivity detected in the meninges using the 6E10 antibody, but minimal reactivity was observed with the 4G8 clone. Several dural blood vessels also showed Aβ immunoreactivity, with a higher frequency observed in sections labeled with the 6E10 clone compared to the 4G8 clone. Notably, staining with the 6E10 clone revealed Aβ immunofluorescence in the walls of dural lymphatic vessels, with 3 out of 6 Alzheimer’s disease (AD) subjects showing Aβ6E10+PDPN+ lymphatic vessels, while none of the 5 control subjects displayed this. In contrast, no immunoreactivity associated with PDPN+ lymphatic vessels was detected when using the 4G8 antibody. As previously noted, the 4G8 antibody effectively labeled cortical Aβ plaques, leptomeningeal Aβ, and Aβ associated with dural arteries. Since the 6E10 antibody specifically detects fibrillary Aβ species, one would expect similar staining patterns with a congophilic dye like Congo red or X-34 if the observed immunoreactivity were specific to Aβ. However, no positive staining with Congo red or X-34 was evident in the meningeal sections of either control or AD subjects. The lack of positive staining with both the 4G8 antibody and congophilic dyes indicates that the anti-Aβ 6E10 immunoreactivity observed in dural tissue from both control and AD subjects, as well as in association with meningeal lymphatic vessels, is likely a result of nonspecific antibody binding rather than a specific localization of Aβ within these structures.

The discovery of lymphatic vessels in the dura mater reinforces the findings of Absinta et al. [73]. The meningeal lymphatic system is conserved across various species, including rodents, nonhuman primates, and humans. The PDPN+LYVE1+CD31− (Type 1) lymphatic vessels identified in this study correspond with peripheral tissue lymphatic capillaries and those described in the murine subarachnoid space [81]. Additionally, PDPN+LYVE1-CD31− (Type 2) dural vessels were found, suggesting a precollector (PDPN+LYVE1-αSMA−) phenotype rather than that of a capillary. Although these structures do not exhibit CD31 staining and are therefore not classified as meningeal blood vessels, it is still possible that the Type 2 structures lined with PDPN+ cells may not be lymphatic. In murine meninges, the subarachnoid space (SSS) is typically flanked by two lymphatic capillaries, while human subjects displayed over five vessels linked to this significantly larger structure. Furthermore, murine meningeal lymphatic vessels generally measure 20–30 μm in diameter [71,81], whereas the human vessels in our study showed a broad diameter range from 19 to 470 μm, supporting the extensive diameter range noted by Absinta et al. [73].

The dura mater, along with several identified dural lymphatic vessels, displayed a diffuse immunoreactivity when stained with the 6E10 antibody clone; however, this reaction was not observed with the 4G8 antibody clone. This outcome was surprising, given that the 4G8 antibody recognizes a wider array of Aβ species, binding both prefibrillar Aβ oligomers and fibrillary Aβ, whereas the 6E10 antibody specifically targets only fibrillary Aβ [83]. Although these antibodies focus on different epitopes within the extracellular domain of the Aβ protein [84], the staining patterns of dense-core Aβ plaques containing fibrillary Aβ are generally comparable [85,86]. To ascertain whether the immunoreactivity observed with 6E10 was attributed to fibrillary Aβ or was nonspecific, we applied Congo Red and X-34 stains to dural samples. Both stains detect fibrillary Aβ independently of antibodies, yielding negative results. These findings suggest that Aβ is not deposited in the dura, indicating that the 6E10-positive labeling was likely nonspecific. This suggests that while interstitial Aβ may exchange into the CSF compartment, it does not significantly deposit within or along the meningeal lymphatic vessels associated with the SSS. This does not imply that these lymphatic vessels do not play a role in the clearance of soluble Aβ from brain tissue; rather, it may simply reflect that Aβ does not specifically deposit along these structures. The relative absence of mural cells surrounding the meningeal lymphatic vasculature, or differences in the physical (e.g., pulsation) or chemical environment (e.g., vessel wall matrix composition) between lymphatic and arterial walls, may impede Aβ associated with these vessels from aggregating as it does in the walls of leptomeningeal or intraparenchymal arteries.

Recent proposals suggest that the distinctive chemical and shear conditions present in the cerebral arterial wall might contribute to the deposition of Aβ associated with CAA [87]. Additionally, tracer studies conducted in both animal models and humans reveal that solutes in the CSF are transported via perineural pathways through the basal cisterns and the cribriform plate [88,89]. Consequently, the meningeal lymphatic vessels in the calvarium may have a limited capacity for Aβ clearance from the CSF when compared to those situated at the base of the skull.

Another rarely reported issue is the involvement of the dural space in AD subjects. This issue has been recently addressed with some interesting findings [90]. The main starting point is the definition of the mechanism of the accumulation of Aβ and tau aggregates within the brain of AD patients, which remains not fully understood [65,91,92,93,94,95]. The impairment of Aβ clearance from the CNS is multifactorial, involving changes in enzymatic degradation [96], transport across the blood–brain and CSF–blood barriers [97], interstitial fluid (ISF) bulk flow [98], and CSF egress [68,70]. CSF is primarily produced in the choroid plexus (ChP), an extension of the ependymal epithelium found in the brain’s ventricles [99]. Most CSF production occurs in the lateral and third ventricles, from where it flows caudally through the aqueduct of Sylvius to the fourth ventricle and then into the subarachnoid space (SAS) via the foramen of Magendie and Luschka. A portion of this fluid subsequently moves into the dural venous sinuses for reabsorption into the bloodstream via arachnoid granulations. Increasing evidence suggests that waste clearance from the CNS may also occur along perivascular and interstitial routes [68]. In this context, CSF flows from the SAS into the periarterial space, transitioning into the interstitial space, facilitated by periarterial aquaporin 4 (AQP4) channels [68,100,101]. It has been suggested that fluid movement within the brain’s parenchymal interstitial space can facilitate the transition of fluid into the perivenous space through AQP4 channels [102]. Recent evaluations of CSF egress, using intrathecally administered contrast agents, have indicated that this may also occur along the parasagittal dural (PSD) space surrounding the sagittal sinus [103,104,105]. Novel MRI techniques allow for the non-invasive quantification of proximal and distal aspects of neurofluid circulation—including ChP function, CSF flow through the aqueduct, and PSD anatomy—in humans [106,107]. Previous studies found age-related hypertrophy in both the ChP and PSD, a decline in ChP perfusion, and reduced caudal CSF flow through the aqueduct of Sylvius. The authors employed dynamic 11C-PIB imaging to quantify Aβ burden, pseudo-continuous arterial spin labeling imaging combined with an F-CNN segmentation method of T1-weighted MPRAGE images for ChP perfusion assessment, and a novel phase-contrast angiography technique to evaluate net CSF flow through the aqueduct of Sylvius and to automatically delineate and quantify the PSD volume on T2-weighted VISTA images. The main finding is that hypertrophy of the total PSD space was significantly correlated with increased global Aβ burden; specifically, hypertrophy in the frontal and parietal subsegments of the PSD was associated with Aβ levels. In contrast, no significant association between ChP perfusion or net CSF flow through the aqueduct of Sylvius and Aβ accumulation was found. These findings imply that the PSD space, previously linked to CSF egress, may play a crucial role in Aβ accumulation. Li et al. [108] demonstrated that impaired ventricular CSF clearance correlates with elevated Aβ concentrations in AD, suggesting that compromised CSF clearance may be a key factor in the disease’s protein aggregation pathology. Furthermore, a stable isotope-labeled kinetic study reported increased turnover of Aβ42, with reduced turnover of Aβ38/40 among amyloid-positive individuals compared to amyloid-negative participants. Under the assumption that blood–brain barrier transport and proteolytic mechanisms for Aβ38/40 and Aβ42 are similarly downregulated, this finding highlights the critical role of CSF clearance mechanisms in Aβ removal [109]. Previous research has indicated a strong aging effect on PSD volume, suggesting a possible compensatory mechanism or evidence of decompensatory tissue dysfunction in response to impaired CSF or ISF drainage with age [106,110]. Notably, the strongest correlation between global Aβ accumulation and PSD volume was observed in the frontal and parietal subsegments. These regions correspond with the typical Aβ aggregation patterns found in the frontal cortex, anterior and posterior cingulate, and precuneus. Additionally, PSD volume was not correlated with overall brain tissue volume, even after adjusting for total intracranial cavity volume, indicating that PSD hypertrophy occurs independently of general brain atrophy. Supporting this, ultrastructural electron microscopy in human dura samples revealed that dural channels were more widely distributed in older patients compared to younger individuals, who exhibited densely concentrated channels around the SSS [105]. These dural channels lacked expression of lymphatic and vascular markers, suggesting a potential reservoir-like function for CSF drainage. Indeed, studies involving intrathecal injection of gadobutrol, an MRI contrast agent serving as a CSF tracer, alongside high-resolution MRI, demonstrated that gadobutrol efflux occurs into the PSD, peaking at 24 h post-injection in humans [103]. In comparison, minimal tracer efflux was noted in the cribriform plate, another hypothesized CSF clearance pathway. Animal studies have shown that complete absence of dural lymphatic vessels results in reduced clearance of macromolecules and abolishes CSF transport from the SAS to deep cervical lymph nodes [71]. Collectively, these findings underscore the important, yet not fully understood, role of the PSD in CSF clearance and egress. One plausible explanation for PSD hypertrophy is that it represents a compensatory mechanism in response to rising Aβ levels, enabling the PSD to enhance clearance by increasing communication with the superior sagittal sinus and/or facilitating collateral paths for CSF drainage along cranial nerves. This could lead to the dilation and/or more diffuse distribution of channels within the frontal and parietal segments of the PSD space, promoting greater CSF efflux. Alternatively, neuroinflammation may serve as a link between Aβ and tau proteinopathies, potentially triggering a hypertrophic response in the PSD [111]. While the mechanisms by which these dural channels around the superior sagittal sinus facilitate CSF egress and waste clearance are still unclear, it is evident that the PSD plays a significant role in CSF drainage, necessitating further characterization of these channels to elucidate their implications in AD and neurodegenerative proteinopathies [111].

## 4. Pathology and Iatrogenic CAA

Neurodegenerative disorders are characterized by a progressive loss of neurons and dysfunction in related systems, often marked by pathological protein deposition in the CNS [112]. Key proteins such as Aβ, tau, α-synuclein, TAR DNA-binding protein TDP-43, and prion protein (PrP) serve as biomarkers for these diseases. A significant concern is the potential for these proteins to transmit disease between individuals, which carries important public health implications. Experimental evidence supports this notion, with a hierarchical framework proposed to strengthen the idea of prion-like transmission of disease-associated proteins [113,114,115,116]. Notably, prion diseases are the only confirmed protein misfolding disorders capable of transmission from human or animal to human. The term “propagon” categorizes this phenomenon into four levels: molecular, tissue, systemic, and infectious propagons [114]. Only prion diseases are believed to meet all these criteria, while evidence for other neurodegenerative proteins is insufficient [114]. Recent studies suggest that iatrogenic Creutzfeldt–Jakob disease (iCJD), linked to cadaveric pituitary hormones [18] or dura mater grafts [117,118], often correlates with Aβ deposition, indicating that Aβ may act as a seed or infectious propagon [114]. Aβ deposition is essential for diagnosing AD, alongside the accumulation of abnormally phosphorylated intracellular tau [119]. Unlike prion diseases, AD is characterized by a gradual decline in cognitive function, with Aβ undergoing a maturation process [120] and following a hierarchical pattern of brain region involvement [121,122].

Two types of CAA are recognized, with three stages of brain involvement proposed [123,124]. Aβ plaques can appear in younger individuals, particularly those carrying the ε4 allele of the apolipoprotein E (APOE) gene [125]. Neuronal phospho-tau pathology may develop in subcortical nuclei during the second decade of life, preceding cortical involvement [126,127,128,129]. Accelerated neurodegeneration has been observed after traumatic brain injury (TBI), whether from a single incident or chronic traumatic encephalopathy (CTE) due to repeated trauma [130,131,132,133,134]. Aβ deposits, along with tau-positive neurofibrillary tangles similar to those seen in AD, are detected following a single TBI [131]. In CTE, tau pathology is predominant, with deposition of other neurodegeneration-related proteins, including Aβ [132,133,135].

A pathological study aimed to investigate whether the Aβ deposition pattern in iCJD from cadaveric dura mater implantation differs from that in AD, cognitively normal young individuals, or TBI; whether dural grafts in iCJD cases exhibit Aβ deposition; and if Aβ accumulates in the dura mater in non-CJD cases [136]. The examined iCJD cases included various brain regions, and both cases showed mild to moderate spongiform changes, gliosis, and neuronal loss with diffuse and synaptic immunoreactivity for disease-associated PrP. Immunostaining revealed parenchymal plaques and CAA, with phosphorylated tau (AT8) staining showing occasional small neuritic profiles but no neurofibrillary tangles or glial tau immunoreactivity.

Histopathological examination revealed that both cases exhibited similar Aβ immunoreactivity morphology, characterized by CAA (type 2) and predominantly focal cortical deposits [118,137,138]. Parenchymal Aβ deposits clustered near surgical operation sites, primarily in the frontal and temporal cortex. Both cases were classified as phase 1 [121], although the involvement of the anterior cingulate in iCJD-2 suggested a potential phase 2 classification. CAA was present in neocortical regions but absent in subcortical areas (stage 1) [124]. Notably, significant tissue damage was observed around the traumatic lesion in iCJD-1, with Aβ exhibiting a lake-like appearance in the adjacent white matter.

In summary, common features observed in the pathological examination of iCJD cases include:

- Amorphous Aβ Deposits: Present in the grafted dura mater but absent in the host dura mater.
- Parenchymal Deposits: Variability in deposits, with more pronounced deposits in iCJD-1 compared to iCJD-2.
- Focal Deposits: Predominance of focal deposits, including mature and immature plaques.
- Location of Plaques: Predominantly adjacent to the traumatic lesion where the graft was implanted.
- Clustering of Plaques: Clustering in cortical areas, lacking a laminar preference.
- Columnar Alignment: Focal deposits aligning columnarly, particularly near the lesion.
- Absence of Tau Pathology: Lack of neuronal or glial tau pathology.

These findings highlight the unique pathological characteristics associated with Aβ deposition in iCJD cases compared to other neurodegenerative conditions like AD.

In the VITA cohort, classic morphology of CAA was found in 11 out of 84 cases (13.09%; median age: 85 years, range 82–89), with amorphous deposits of Aβ identified in connective tissue adjacent to dural sinuses in 11 cases (13.09%; median age: 86 years, range 84–88). The age distribution showed no significant differences between groups with and without Aβ immunoreactivity in the dura. Both CAA and amorphous deposits were detectable using various antibodies. The combination of CAA and amorphous deposits occurred in only five cases, and enhanced proteinase K treatment allowed detection of both lesion types, which exhibited birefringence in Congo staining. Notably, cases with typical CAA exhibited Aβ deposits in the brain (100%), with dural amorphous deposits significantly associated with Aβ deposits in the brain (100%) [139].

In sporadic AD, Aβ deposits are classified as diffuse, stellate, and focal [119]. A grading system suggests a progression from purely focal Aβ deposition to the emergence of Congo red positive material, followed by immunoreactivity for hyperphosphorylated tau [120]. Diffuse Aβ deposits are recognized as the earliest form, with mature plaques appearing later [114,119]. However, the two iCJD cases exhibited a unique Aβ deposition pattern, lacking a clear laminar arrangement or homogeneous cortical involvement.

Increased Aβ pathology frequency in our iCJD cases may be attributed to brain trauma. Although recent studies indicate that repetitive injury can lead to CTE, the examined patients showed no documentation of repeated trauma or clinical symptoms indicative of CTE [136]. The plaque distribution exhibited higher density near the dura mater graft.

In summary, the pathology observed in the iCJD cases was distinct from that seen in AD brains and long-term TBI or CTE survivors. The identification of Aβ deposits in the grafted dura, but not in the host dura mater, suggests a scenario where pathological protein seeds into the underlying CNS. However, Aβ seeds alone seem insufficient to replicate the complete clinicopathological phenotype of AD [84,125,129,130,131,133,135,140,141,142,143,144,145,146,147,148,149].

Despite the small size of the dura sample examined (4 cm^2^), Aβ deposits associated with AD pathology were detected within the CNS [136]. Both CAA and amorphous deposits were labeled by all antibodies, exhibiting birefringence in Congo red staining. It has been suggested that amyloid proteins have a ubiquitous affinity for basement membranes [150], as evidenced by dura-associated Aβ deposits. Recent studies confirmed the presence of a lymphatic system lining the dural sinuses, which drains the brain’s interstitial fluid [30,71,72,151,152,153,154,155]. This finding may help explain the accumulation of amorphous Aβ deposits near dural sinuses in our cohort of elderly individuals. The main information about the dural pathology in CAA and iCJD is summarized in Table 1.

## 5. Subdural Hematoma and CAA

CAA is a common cause of spontaneous intracerebral hemorrhage (ICH) in elderly patients, characterized by the deposition of amyloid-beta (Aβ) in the walls of small-caliber cortical and leptomeningeal vessels. Recent studies indicate a potential association between CAA and subdural hematoma (SDH), which has not been clearly established in the previous literature.

Xia et al. [156] conducted a study involving 98 patients with CAA-related ICH, of whom 35 had associated SDH. The study found that patients in the SDH group had significantly higher rates of postoperative hemorrhage and worse surgical outcomes compared to those without SDH. These findings suggest that the presence of SDH may indicate a more complex pathology and worse prognosis in patients with CAA. Zupan et al. [157] presented a case series of three patients who experienced acute lobar ICH accompanied by SDH. Histopathological analysis confirmed CAA in all cases, highlighting that SDH may be a more frequent manifestation of CAA than previously recognized. This underscores the need for clinicians to consider SDH in patients with CAA presenting with neurological deficits. Bruce et al. [158] reported a case of a 77-year-old woman where SDH presented as an initial symptom of biopsy-proven CAA-related inflammation. This case further supports the notion that CAA can lead to SDH without significant prior trauma or ICH. Andres et al. [159] conducted a large retrospective cohort study that demonstrated a significant association between CAA and isolated SDH. Their findings indicated that patients with CAA had a threefold increased risk of developing SDH compared to individuals with other cerebrovascular diseases, thus establishing CAA as a potential risk factor for isolated SDH. Rivier et al. [160] also found that in two large cohorts, CAA was associated with an increased risk of isolated non-traumatic SDH, confirming the findings of earlier studies. The association was significant even after adjusting for various risk factors, suggesting that CAA could independently contribute to the development of SDH.

**Figure 1:**
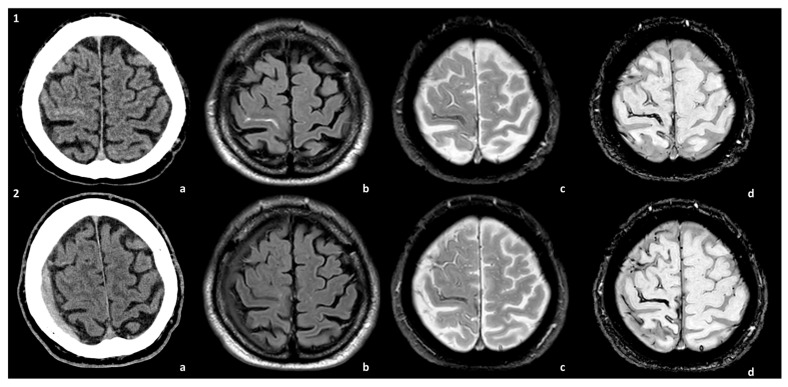
Spontaneous SDH in the natural history of a patient with CAA. The patient presented with TFNEs and convexal subarachnoid hemorrhage with cortical superficial siderosis (cSS) (**panel 1**). A right hemispheric SDH was found in the control MRI at 1 year (**panel 2**). The images are compared at the two time points of the natural history of the patient: (**a**) non-contrast CT, (**b**) axial Fluid-Attenuated Inversion Recovery (FLAIR) sequence, (**c**) T2* sequence, and (**d**) Susceptibility-Weighted Imaging (SWI) sequence.

**Figure 2:**
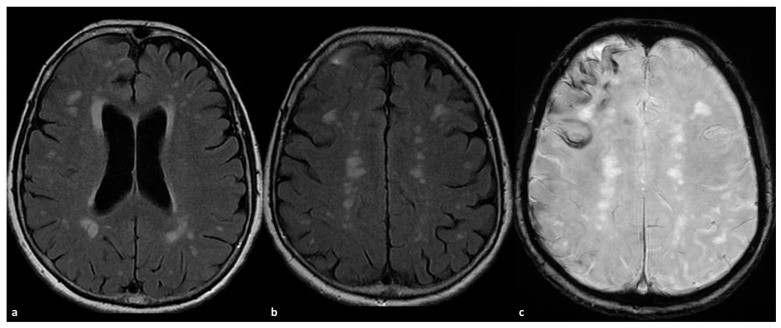
CAA-related inflammation with prominent sulcal effacement and gyral swelling in the right frontal pole ((**a**,**b**), axial FLAIR) with simultaneous pachymeningeal involvement and small satellite subdural collection (**a**,**b**). The patient has a diffuse cSS ((**c**) SWI) with clustering on the right frontal pole.

Some examples of the association of SDH with CAA and CAA-related inflammation are proposed in Figure 1 and Figure 2.

The underlying mechanisms linking CAA and SDH may include:

- **Vascular Fragility**: The amyloid deposition in vessel walls may lead to a structural weakness, increasing the likelihood of vascular rupture, particularly during periods of increased intracranial pressure or minor trauma.
- **Cerebral Atrophy**: Age-related brain atrophy, common in CAA patients, may stretch and rupture bridging veins, resulting in SDH.
- **Inflammatory Processes**: CAA-related inflammation may contribute to the development of fragile capillaries, which could rupture and lead to SDH.

The association between CAA and subdural hematoma is increasingly recognized and signifies a complex interplay of vascular pathology in aging populations. The presence of SDH in patients with CAA may indicate a worse prognosis and necessitates a reevaluation of management strategies for these patients. However, at the moment, no information is available about a treatment different from the usual one for SDH in patients with CAA, and even less is known about the potential role of SDH as driver of ICH or convexal SAH risk and management in these patients. Future research should focus on establishing clear causal relationships and potential therapeutic interventions to mitigate risks associated with these conditions.

## 6. Cerebral Amyloid Angiopathy: Pathological Side of Vascular Contribution to Alzheimer’s Disease

As extensively documented in the literature, the primary comorbidity associated with CAA is AD, particularly from a histopathological perspective. The prevalence of this association highlights the potential role of vascular amyloid in cognitive decline and the presence of vascular comorbidities in AD patients, evident in MRI findings of SVD as well as in clinical manifestations, including hemorrhagic and ischemic events. Both CAA and AD are progressive conditions, exhibiting a clear temporal impact on the extent of vascular damage. Autopsy studies reveal a prevalence rate of approximately 5–9% in individuals aged 60–69, escalating to around 43–58% in those over 90 years old [161,162]. Moreover, postmortem evaluations of AD patients indicate the presence of CAA in 90% of cases [1]. It is estimated that 20–40% of cognitively intact individuals and 50–60% of those diagnosed with a neurocognitive disorder over the age of 80 exhibit CAA [1]. Genetic factors play a significant role as non-modifiable risk factors for CAA [163]. Specific alleles of the apolipoprotein E (ApoE) gene are linked to sporadic instances of both CAA and AD. In the context of AD, the ε4 allele is recognized as the most substantial genetic risk factor. Carriers of the ε4 allele are at an increased risk for developing AD and often experience an earlier onset, correlating with higher levels of Aβ plaques in the brain. It is suggested that the ε4 allele influences Aβ clearance and promotes its aggregation, contributing to plaque formation and neurodegeneration in AD [163]. A larger cohort of pathologically confirmed CAA cases analyzed for AD pathology has revealed an unexpectedly high prevalence of the ɛ2 allele [164]. In terms of CAA, both the ε4 and ε2 alleles of the ApoE gene are linked to an increased risk. Carriers of the ε4 allele are more likely to develop CAA, often experiencing more severe forms of the disease. Conversely, while the ε2 allele is associated with a heightened risk of CAA, it is to a lesser degree than the ε4 allele [165,166]. Nonetheless, the precise mechanisms through which specific ApoE alleles influence the development and progression of AD and CAA are still under investigation. It is believed that the ApoE genotype affects Aβ metabolism and clearance, amyloid plaque deposition, and the integrity of cerebral blood vessels [33,167,168,169].

From a pathological viewpoint, the primary characteristics include the load of amyloid-beta within the walls of arterioles, capillaries, and, to a lesser extent, venules in the cerebral cortex and leptomeninges. Despite the known association between CAA and hemorrhagic risk, pathologically severe CAA can exist without lobar hemorrhages [170,171]. Depending on the extent of vessel wall damage, CAA can be histologically categorized as mild, moderate, or severe [171]. In most AD cases, the damage is microscopically mild, characterized by amyloid deposition replacing vascular smooth muscle cells or pericytes, along with thickening of the vessel wall. However, secondary changes may occur, including various types of inflammation, aneurysmal dilation, and bleeding. Reports linking AD or age-related CAA with secondary vascular changes are rare [171,172], classified as: (1) hyalinization/fibrosis, (2) smooth muscle cell degeneration, (3) ‘double-barreling’ phenomenon, (4) macrophage infiltration within the vessel wall, (5) multinucleated giant cells in or around the vessel wall, (6) perivascular chronic inflammation, (7) calcification, (8) necrosis of the vessel wall, (9) microaneurysms, (10) recent microhemorrhages, and (11) iron and hemosiderin deposition [173]. Accumulation of amyloid-beta in the vascular tunica media and/or adventitia is associated with the degeneration of endothelial cells [174,175], vascular smooth muscle cells [176], and pericytes [177], ultimately compromising the integrity of the blood–brain barrier and potentially leading to defects in the vessel wall [178]. Hyalinized vessels often exhibit weak amyloid-beta immunoreactivity, indicating that over time, vascular amyloid leads to the degeneration of vascular cells, gradually replaced by hyalinized fibrous tissue in the vessel wall [179]. Vessels with significant amyloid deposition in the tunica media and adventitia display the double-barreling phenomenon [171], where amyloid positivity is frequently absent in the remnants of the inner vessel wall. This appearance may result from the loss of smooth muscle cells, leading to the splitting of the tunica media or separation from the tunica adventitia. Additionally, CAA is associated with fibrinoid necrosis and the formation of microaneurysms. Ultimately, these degenerative changes can impair vessel wall integrity, culminating in rupture and cerebral hemorrhages [179]. Currently, inflammation is viewed as a major contributor to damage in CAA, associated with spontaneous manifestations in CAA patients, referred to as CAA-related inflammation [11,180,181,182,183,184], as well as infrequent spontaneous and more commonly drug-induced events in AD patients undergoing immunotherapy (Amyloid-related Imaging Abnormalities or ARIA-E) [185,186,187].

Numerous components of the amyloid cascade and their mechanisms of vascular and neuronal damage have been outlined, yet a comprehensive theory explaining the pathogenesis of both CAA and AD remains elusive. The involvement of the dura mater in CAA, as well as the role of the venular component in conjunction with arterial involvement [64] and the impact of traumatic brain injury on CAA [134], are areas that warrant further exploration. Additional studies and evidence are essential to clarify these complex interactions and address the vascular contributions to AD and dementia from both preventive and therapeutic perspectives.

## 7. Conclusions

In conclusion, the investigation into the mechanisms of dural involvement in CAA reveals a complex interplay between vascular pathology, amyloid deposition, and the implications for neurodegenerative processes. While CAA is primarily characterized by Aβ accumulation in small vessels, emerging evidence suggests that the dura mater and its associated lymphatic system may also play a significant role in the disease’s pathology. Recent studies indicate that the dura mater is not merely an inert structure but may actively participate in the clearance of interstitial fluid and Aβ, potentially influencing the progression of CAA. The identification of meningeal lymphatic vessels in both rodent models and humans underscores the importance of the dural lymphatic system in maintaining cerebrospinal fluid homeostasis and facilitating the clearance of toxic metabolites. This suggests that impaired drainage through these lymphatics could contribute to the pathological accumulation of Aβ, exacerbating CAA and its neurological consequences.

Moreover, the relationship between CAA and SDH highlights the need for a nuanced understanding of vascular fragility and the inflammatory processes that may predispose individuals to hemorrhagic events. The presence of SDH in patients with CAA not only complicates the clinical picture but also indicates a potentially worse prognosis, warranting careful monitoring and management strategies.

As research continues to elucidate the mechanisms underlying dural involvement, it becomes increasingly clear that a multidisciplinary approach, integrating findings from neuropathology, neuroimaging, and clinical investigations, is essential. This comprehensive understanding will pave the way for novel therapeutic strategies aimed at mitigating the impact of CAA and improving outcomes for affected individuals.

In summary, recognizing the significance of dural involvement expands our understanding of CAA and its clinical manifestations, emphasizing the necessity for further research into the dural lymphatic system and its role in cerebrovascular health. Future studies should focus on the interactions between amyloid pathology, vascular integrity, and lymphatic drainage, as these factors collectively influence the trajectory of CAA and its associated cognitive decline.

## Figures and Tables

**Table 1:** Main features of dural deposits of amyloid-beta.

Presence and Characteristics	Amyloid deposits were identified in the dura mater (pachymeninx) adjacent to dural sinuses in a subset of cases, demonstrating a distinct accumulation of Aβ without selectivity for Aβ1–40 or Aβ1–42 in amorphous deposits. These deposits were detected using various antibodies, including 4G8, 6F/3D, anti-Aβ1–40, and anti-Aβ1–42, and exhibited birefringence under polarized light with Congo red staining.
Association with Other Conditions	Dural amyloid deposits were noted in cases of generalized amyloidosis and were linked to areas where the blood–brain barrier is compromised. In the context of iCJD (iatrogenic Creutzfeldt–Jakob disease), Aβ deposits in the grafted dura mater were absent in the host dura mater, suggesting a potential mechanism where pathological proteins seed into the underlying CNS.
Histopathological Findings	The study highlighted the presence of amorphous Aβ deposits in the grafted dura, which were not found in the surrounding host dura mater. Collagen IV was noted in the connective tissue where Aβ immunoreactivity occurred, marking basement membranes.
Lymphatic System	Recent studies confirmed the presence of a lymphatic system lining the dural sinuses, which drains interstitial fluid from the brain. This finding may explain the accumulation of amorphous Aβ deposits near the dural sinuses in elderly individuals.
Implications	The findings suggest that the dura mater, traditionally considered metabolically inert and avascular, plays a role in amyloid pathology and may contribute to conditions such as CAA due to impaired clearance or increased Aβ production.

## Data Availability

No new data were produced in this paper.

## Abbreviations

The following abbreviations are used in this manuscript:

AD	Alzheimer’s Disease
BBB	Blood–Brain Barrier
CAA	Cerebral Amyloid Angiopathy
CNS	Central Nervous System
CSF	Cerebrospinal Fluid
CTE	Chronic Traumatic Encephalopathy
ICH	Intracerebral hemorrhage
iCJD	Iatrogenic Creutzfeldt–Jakob Disease
MRI	Magnetic Resonance Angiography
SDH	Subdural Hematoma
TBI	Traumatic Brain Injury

## References

[1] Jakel L., De Kort A.M., Klijn C.J.M., Schreuder F., Verbeek M.M. (2021). Prevalence of cerebral amyloid angiopathy: A systematic review and meta-analysis. Alzheimer’s Dement..

[2] Samarasekera N., Smith C., Al-Shahi Salman R. (2012). The association between cerebral amyloid angiopathy and intracerebral haemorrhage: Systematic review and meta-analysis. J. Neurol. Neurosurg. Psychiatry.

[3] Flaherty M.L., Haverbusch M., Sekar P., Kissela B., Kleindorfer D., Moomaw C.J., Sauerbeck L., Schneider A., Broderick J.P., Woo D. (2006). Long-term mortality after intracerebral hemorrhage. Neurology.

[4] Charidimou A., Imaizumi T., Moulin S., Biffi A., Samarasekera N., Yakushiji Y., Peeters A., Vandermeeren Y., Laloux P., Baron J.-C. (2017). Brain hemorrhage recurrence, small vessel disease type, and cerebral microbleeds: A meta-analysis. Neurology.

[5] Smith E.E., Charidimou A., Ayata C., Werring D.J., Greenberg S.M. (2021). Cerebral amyloid angiopathy-related transient focal neurologic episodes. Neurology.

[6] Boyle P.A., Yu L., Wilson R.S., Leurgans S.E., Schneider J.A., Bennett D.A. (2018). Person-specific contribution of neuropathologies to cognitive loss in old age. Ann. Neurol..

[7] Gokcal E., Horn M.J., van Veluw S.J., Frau-Pascual A., Das A.S., Pasi M., Fotiadis P., Warren A.D., Schwab K., Rosand J. (2021). Lacunes, microinfarcts, and vascular dysfunction in cerebral amyloid angiopathy. Neurology.

[8] Reijmer Y.D., Fotiadis P., Martinez-Ramirez S., Salat D.H., Schultz A., Shoamanesh A., Ayres A.M., Vashkevich A., Rosas D., Schwab K. (2015). Structural network alterations and neurological dysfunction in cerebral amyloid angiopathy. Brain.

[9] McCreary C.R., Beaudin A.E., Subotic A., Zwiers A.M., Alvarez A., Charlton A., Goodyear B.G., Frayne R., Smith E.E. (2020). Cross-sectional and longitudinal differences in peak skeletonized white matter mean diffusivity in cerebral amyloid angiopathy. Neuroimage Clin..

[10] Raposo N., Zanon Zotin M.C., Schoemaker D., Xiong L., Fotiadis P., Charidimou A., Pasi M., Boulouis G., Schwab K., Schirmer M.D. (2021). Peak width of skeletonized mean diffusivity as neuroimaging biomarker in cerebral amyloid angiopathy. AJNR Am. J. Neuroradiol..

[11] Charidimou A., Boulouis G., Frosch M.P., Baron J.-C., Pasi M., Albucher J.F., Banerjee G., Barbato C., Bonneville F., Brandner S. (2022). The Boston criteria version 2.0 for cerebral amyloid angiopathy: A multicentre, retrospective, MRI-neuropathology diagnostic accuracy study. Lancet Neurol..

[12] Rodrigues M.A., Samarasekera N., Lerpiniere C., Humphreys C., McCarron M.O., White P.M., Nicoll J.A.R., Sudlow C.L.M., Cordonnier C., Wardlaw J.M. (2018). The Edinburgh CT and genetic diagnostic criteria for lobar intracerebral haemorrhage associated with cerebral amyloid angiopathy: Model development and diagnostic test accuracy study. Lancet Neurol..

[13] Rodrigues M.A., Seiffge D., Samarasekera N., Moullaali T.J., Wardlaw J.M., Schreiber S., Behymer T.P., Khandwala V., Stanton R.J., Vagal V. (2025). Association between the Edinburgh CT and genetic diagnostic criteria for cerebral amyloid angiopathy-associated lobar intracerebral haemorrhage and recurrent intracerebral haemorrhage: An individual patient data meta-analysis. Lancet Neurol..

[14] van Etten E.S., Gurol M.E., van der Grond J., Haan J., Viswanathan A., Schwab K.M., Ayres A.M., Algra A., Rosand J., Van Buchem M.A. (2016). Recurrent hemorrhage risk and mortality in hereditary and sporadic cerebral amyloid angiopathy. Neurology.

[15] Banerjee G., Carare R., Cordonnier C., Greenberg S.M., Schneider J.A., Smith E.E., van Buchem M., van der Grond J., Verbeek M.M., Werring D.J. (2017). The increasing impact of cerebral amyloid angiopathy: Essential new insights for clinical practice. J. Neurol. Neurosurg. Psychiatry.

[16] Dumas A., Dierksen G.A., Gurol M.E., Halpin A., Martinez-Ramirez S., Schwab K., Rosand J., Viswanathan A., Salat D.H., Polimeni J.R. (2012). Functional magnetic resonance imaging detection of vascular reactivity in cerebral amyloid angiopathy. Ann. Neurol..

[17] van Opstal A.M., van Rooden S., van Harten T., Ghariq E., Labadie G., Fotiadis P., Gurol M.E., Terwindt G.M., Wermer M.J., van Buchem M.A. (2017). Cerebrovascular function in presymptomatic and symptomatic individuals with hereditary cerebral amyloid angiopathy: A case-control study. Lancet Neurol..

[18] Jaunmuktane Z., Mead S., Ellis M., Wadsworth J.D.F., Nicoll A.J., Kenny J., Launchbury F., Linehan J., Richard-Loendt A., Walker A.S. (2015). Evidence for human transmission of amyloid-βpathology and cerebral amyloid angiopathy. Nature.

[19] Greenberg S.M., Bacskai B.J., Hernandez-Guillamon M., Pruzin J., Sperling R., van Veluw S.J. (2020). Cerebral amyloid angiopathy and Alzheimer disease—One peptide, two pathways. Nat. Rev. Neurol..

[20] Wardlaw J.M., Smith C., Dichgans M. (2019). Small vessel disease: Mechanisms and clinical implications. Lancet Neurol..

[21] Uekawa K., Hattori Y., Ahn S.J., Seo J., Casey N., Anfray A., Zhou P., Luo W., Anrather J., Park L. (2023). Border-associated macrophages promote cerebral amyloid angiopathy and cognitive impairment through vascular oxidative stress. Mol. Neurodegener..

[22] Koemans E.A., Chhatwal J.P., van Veluw S.J., van Etten E.S., van Osch M.J.P., van Walderveen M.A.A., Sohrabi H.R., Kozberg M.G., Shirzadi Z., Terwindt G.M. (2023). Progression of cerebral amyloid angiopathy: A pathophysiological framework. Lancet Neurol..

[23] Schultz A.P., Kloet R.W., Sohrabi H.R., van der Weerd L., van Rooden S., Wermer M.J.H., Moursel L.G., Yaqub M., van Berckel B.N.M., Chatterjee P. (2019). Amyloid imaging of dutch-type hereditary cerebral amyloid angiopathy carriers. Ann. Neurol..

[24] Bateman R.J., Xiong C., Benzinger T.L., Fagan A.M., Goate A., Fox N.C., Marcus D.S., Cairns N.J., Xie X., Blazey T.M. (2012). Clinical biomarker changes in dominantly inherited Alzheimer’s disease. N. Engl. J. Med..

[25] Chatterjee P., Tegg M., Pedrini S., van der Weerd L., van Rooden S., Wermer M.J.H., Moursel L.G., Yaqub M., van Berckel B.N.M., Chatterjee P. (2021). Plasma amyloid-beta levels in a pre-symptomatic dutch-type hereditary cerebral amyloid angiopathy pedigree: A cross-sectional and longitudinal investigation. Int. J. Mol. Sci..

[26] Strozyk D., Blennow K., White L.R., Launer L.J. (2003). CSF Abeta 42 levels correlate with amyloid-neuropathology in a population-based autopsy study. Neurology.

[27] Wiltfang J., Esselmann H., Bibl M., Hüll M., Hampel H., Kessler H., Frölich L., Schröder J., Peters O., Jessen F. (2007). Amyloid beta peptide ratio 42/40 but not A beta 42 correlates with phospho-Tau in patients with low-and high-CSF A beta 40 load. J. Neurochem..

[28] Purro S.A., Farrow M.A., Linehan J., Nazari T., Thomas D.X., Chen Z., Mengel D., Saito T., Saido T., Rudge P. (2018). Transmission of amyloid-βprotein pathology from cadaveric pituitary growth hor-mone. Nature.

[29] Banerjee G., Adams M.E., Jaunmuktane Z., Lammie G.A., Turner B., Wani M., Sawhney I.M.S., Houlden H., Mead S., Brandner S. (2019). Early onset cerebral amyloid angiopathy following childhood exposure to cadaveric dura. Ann. Neurol..

[30] Keable A., Fenna K., Yuen H.M., Johnston D.A., Smyth N.R., Smith C., Salman R.A.-S., Samarasekera N., Nicoll J.A., Attems J. (2016). Deposition of amyloid βin the walls of human leptomeningeal arteries in relation to perivascular drainage pathways in cerebral amyloid angiopathy. Biochim. Biophys. Acta.

[31] Domnitz S.B., Robbins E.M., Hoang A.W., Garcia-Alloza M., Hyman B.T., Rebeck G.W., Greenberg S.M., Bacskai B.J., Frosch M.P. (2005). Progression of cerebral amyloid angiopathy in transgenic mouse models of Alzheimer disease. J. Neuropathol. Exp. Neurol..

[32] Robbins E.M., Betensky R.A., Domnitz S.B., Purcell S.M., Garcia-Alloza M., Greenberg C., Rebeck G.W., Hyman B.T., Greenberg S.M., Frosch M.P. (2006). Kinetics of Cerebral Amyloid Angiopathy Progression in a Transgenic Mouse Model of Alzheimer Disease. J. Neurosci..

[33] Rannikmäe K., Kalaria R.N., Greenberg S.M., Chui H.C., Schmitt F.A., Samarasekera N., Salman R.A.-S., Sudlow C.L.M. (2013). APOE associations with severe CAA-associated vasculopathic changes: Collaborative meta-analysis. J. Neurol. Neurosurg. Psychiatry.

[34] Smith E.E., Vijayappa M., Lima F., Delgado P., Wendell L., Rosand J., Greenberg S.M. (2008). Impaired visual evoked flow velocity response in cerebral amyloid angiopathy. Neurology.

[35] Switzer A.R., McCreary C., Batool S., Stafford R.B., Frayne R., Goodyear B.G., Smith E.E. (2016). Longitudinal decrease in blood oxygenation level dependent response in cerebral amyloid angiopathy. NeuroImage Clin..

[36] van Dijk S.E., van der Grond J., Lak J., van den Berg-Huysmans A., Labadie G., Terwindt G.M., Wermer M.J.H., Gurol M.E., van Buchem M.A., Greenberg S.M. (2022). Longitudinal Progression of Magnetic Resonance Imaging Markers and Cognition in Dutch-Type Hereditary Cerebral Amyloid Angiopathy. Stroke.

[37] Shin H.K., Jones P.B., Garcia-Alloza M., Borrelli L., Greenberg S.M., Bacskai B.J., Frosch M.P., Hyman B.T., Moskowitz M.A., Ayata C. (2007). Age-dependent cerebrovascular dysfunction in a transgenic mouse model of cerebral amyloid angiopathy. Brain.

[38] van Veluw S.J., Hou S.S., Calvo-Rodriguez M., Arbel-Ornath M., Snyder A.C., Frosch M.P., Greenberg S.M., Bacskai B.J. (2020). Vasomotion as a Driving Force for Paravascular Clearance in the Awake Mouse Brain. Neuron.

[39] Hawkes C.A., Härtig W., Kacza J., Schliebs R., Weller R.O., Nicoll J.A., Carare R.O. (2011). Perivascular drainage of solutes is impaired in the ageing mouse brain and in the presence of cerebral amyloid angiopathy. Acta Neuropathol..

[40] Arbel-Ornath M., Hudry E., Eikermann-Haerter K., Hou S., Gregory J.L., Zhao L., Betensky R.A., Frosch M.P., Greenberg S.M., Bacskai B.J. (2013). Interstitial fluid drainage is impaired in ischemic stroke and Alzheimer’s disease mouse models. Acta Neuropathol..

[41] Kim S.H., Ahn J.H., Yang H., Lee P., Koh G.Y., Jeong Y. (2020). Cerebral amyloid angiopathy aggravates perivascular clearance impairment in an Alzheimer’s disease mouse model. Acta Neuropathol. Commun..

[42] Aldea R., Weller R.O., Wilcock D.M., Carare R.O., Richardson G. (2019). Cerebrovascular smooth muscle cells as the drivers of intramural periarterial drainage of the brain. Front. Aging Neurosci..

[43] Kedarasetti R.T., Drew P.J., Costanzo F. (2022). Arterial vasodilation drives convective fluid flow in the brain: A poroelastic model. Fluids Barriers CNS.

[44] van den Brink H., Zwiers A., Switzer A.R., Charlton A., McCreary C.R., Goodyear B.G., Frayne R., Biessels G.J., Smith E.E. (2018). Cortical microinfarcts on 3T magnetic resonance imaging in cerebral amyloid angiopathy. Stroke.

[45] Gokcal E., Horn M.J., Becker J.A., Das A.S., Schwab K., Biffi A., Rost N., Rosand J., Viswanathan A., Polimeni J.R. (2022). Effect of vascular amyloid on white matter disease is mediated by vascular dysfunction in cerebral amyloid angiopathy. J. Cereb. Blood Flow Metab..

[46] Shirzadi Z., Yau W.W., Schultz S.A., Schultz A.P., Scott M.R., Goubran M., Mojiri-Forooshani P., Joseph-Mathurin N., Kantarci K., Preboske G. (2022). Progressive White Matter Injury in Preclinical Dutch Cerebral Amyloid Angiopathy. Ann. Neurol..

[47] van Rooden S., van Opstal A.M., Labadie G., Terwindt G.M., Wermer M.J., Webb A.G., Middelkoop H.A., Greenberg S.M., Van Der Grond J., Van Buchem M.A. (2016). Early magnetic resonance imaging and cognitive markers of hereditary cerebral amyloid angiopathy. Stroke.

[48] van Veluw S.J., Reijmer Y.D., van der Kouwe A.J., Charidimou A., Riley G.A., Leemans A., Bacskai B.J., Frosch M.P., Viswanathan A., Greenberg S.M. (2019). Histopathology of diffusion imaging abnormalities in cerebral amyloid angiopathy. Neurology.

[49] Suter O.-C., Sunthorn T., Kraftsik R., Straubel J., Darekar P., Khalili K., Miklossy J. (2002). Cerebral Hypoperfusion Generates Cortical Watershed Microinfarcts in Alzheimer Disease. Stroke.

[50] van Veluw S.J., Scherlek A.A., Freeze W.M., ter Telgte A., van der Kouwe A.J., Bacskai B.J., Frosch M.P., Greenberg S.M. (2019). Different microvascular alterations underlie microbleeds and microinfarcts. Ann. Neurol..

[51] Charidimou A., Hong Y.T., Jäger H.R., Fox Z., Aigbirhio F.I., Fryer T.D., Menon D.K., Warburton E.A., Werring D.J., Baron J.C. (2015). White matter perivascular spaces on magnetic resonance imaging: Marker of cerebrovascular amyloid burden?. Stroke.

[52] Tsai H.H., Pasi M., Tsai L.K., Huang C.-C., Chen Y.-F., Lee B.-C., Yen R.-F., Gurol M.E., Jeng J.-S. (2021). Centrum semiovale perivascular space and amyloid deposition in spontaneous intracerebral hemorrhage. Stroke.

[53] Greenberg S.M., Nandigam R.N., Delgado P., Betensky R.A., Rosand J., Viswanathan A., Frosch M.P., Smith E.E. (2009). Microbleeds versus macrobleeds: Evidence for distinct entities. Stroke.

[54] Pasi M., Marini S., Morotti A., Boulouis G., Xiong L., Charidimou A., Ayres A., Lee M.J., Biffi A., Goldstein J.N. (2018). Abstract 133: Cerebellar Hematoma Location: Implications for the Underlying Microangiopathy. Stroke.

[55] Greenberg S.M., Vernooij M.W., Cordonnier C., Viswanathan A., Salman R.A.-S., Warach S., Launer L.J., Van Buchem M.A., Breteler M.M. (2009). Cerebral microbleeds: A guide to detection and interpretation. Lancet Neurol..

[56] Charidimou A., Linn J., Vernooij M.W., Opherk C., Akoudad S., Baron J.-C., Greenberg S.M., Jäger H.R., Werring D.J. (2015). Cortical superficial siderosis: Detection and clinical significance in cerebral amyloid angiopathy and related conditions. Brain.

[57] Koemans E.A., Voigt S., Rasing I., van Harten T.W., Jolink W.M., Schreuder F.H., van Zwet E.W., van Buchem M.A., van Osch M.J., Terwindt G.M. (2022). Cerebellar Superficial Siderosis in Cerebral Amyloid Angiopathy. Stroke.

[58] Reuter B., Venus A., Heiler P., Schad L., Ebert A., Hennerici M.G., Grudzenski S., Fatar M. (2016). Development of Cerebral Microbleeds in the APP23-Transgenic Mouse Model of Cerebral Amyloid Angiopathy—A 9.4 Tesla MRI Study. Front. Aging Neurosci..

[59] Marazuela P., Paez-Montserrat B., Bonaterra-Pastra A., Solé M., Hernández-Guillamon M. (2022). Impact of cerebral amyloid angiopathy in two transgenic mouse models of cerebral β-amyloidosis: A neuropathological study. Int. J. Mol. Sci..

[60] Kozberg M.G., Yi I., Freeze W.M., Auger C.A., Scherlek A.A., Greenberg S.M., van Veluw S.J. (2022). Blood–brain barrier leakage and perivascular inflammation in cerebral amyloid angiopathy. Brain Commun..

[61] Blevins B.L., Vinters H.V., Love S., Wilcock D.M., Grinberg L.T., Schneider J.A., Kalaria R.N., Katsumata Y., Gold B.T., Wang D.J.J. (2020). Brain arteriolosclerosis. Acta Neuropathol..

[62] Charidimou A., Perosa V., Frosch M.P., Scherlek A.A., Greenberg S.M., van Veluw S.J. (2020). Neuropathological correlates of cortical superficial siderosis in cerebral amyloid angiopathy. Brain.

[63] Charidimou A., Zonneveld H.I., Shams S., Kantarci K., Shoamanesh A., Hilal S., Yates P.A., Boulouis G., Na H.K., Pasi M. (2019). APOE and cortical superficial siderosis in CAA: Meta-analysis and potential mechanisms. Neurology.

[64] Zedde M., Grisendi I., Assenza F., Vandelli G., Napoli M., Moratti C., Lochner P., Seiffge D.J., Piazza F., Valzania F. (2023). The Venular Side of Cerebral Amyloid Angiopathy: Proof of Concept of a Neglected Issue. Biomedicines.

[65] Mawuenyega K.G., Sigurdson W., Ovod V., Munsell L., Kasten T., Morris J.C., Yarasheski K.E., Bateman R.J. (2010). Decreased clearance of CNS beta-amyloid in Alzheimer’s disease. Science.

[66] Patterson B.W., Elbert D.L., Mawuenyega K.G., Kasten T., Ovod V., Ma S., Xiong C., Chott R., Yarasheski K., Sigurdson W. (2015). Age and amyloid effects on human central nervous system amyloid-beta kinetics. Ann. Neurol..

[67] Iliff J.J., Goldman S.A., Nedergaard M. (2015). Implications of the discovery of brain lymphatic pathways. Lancet Neurol..

[68] Iliff J.J., Wang M., Liao Y., Plogg B.A., Peng W., Gundersen G.A., Benveniste H., Vates G.E., Deane R., Goldman S.A. (2012). A Paravascular pathway facilitates CSF flow through the brain Parenchyma and the clearance of interstitial solutes, including Amyloid. Sci. Transl. Med..

[69] Ramanathan A., Nelson A.R., Sagare A.P., Zlokovic B.V. (2015). Impaired vascular mediated clearance of brain amyloid beta in Alzheimer’s disease: The role, regulation and restoration of LRP1. Front. Aging Neurosci..

[70] Tarasoff-Conway J.M., Carare R.O., Osorio R.S., Glodzik L., Butler T., Fieremans E., Axel L., Rusinek H., Nicholson C., Zlokovic B.V. (2015). Clearance systems in the brain-implications for Alzheimer disease. Nat. Rev. Neurol..

[71] Aspelund A., Antila S., Proulx S.T., Karlsen T.V., Karaman S., Detmar M., Wiig H., Alitalo K. (2015). A dural lymphatic vascular system that drains brain interstitial fluid and macromolecules. J. Exp. Med..

[72] Louveau A., Smirnov I., Keyes T.J., Eccles J.D., Rouhani S.J., Peske J.D., Derecki N.C., Castle D., Mandell J.W., Lee K.S. (2015). Structural and functional features of central nervous system lymphatic vessels. Nature.

[73] Absinta M., Ha S.-K., Nair G., Sati P., Luciano N.J., Palisoc M., Louveau A., Zaghloul K.A., Pittaluga S., Kipnis J. (2017). Human and nonhuman primate meninges harbor lymphatic vessels that can be visualized noninvasively by MRI. Elife.

[74] Boulton M., Flessner M., Armstrong D., Mohamed R., Hay J., Johnston M. (1999). Contribution of extracranial lymphatics and arachnoid villi to the clearance of a CSF tracer in the rat. Am. J. Physiol..

[75] Bradbury M.W., Cole D.F. (1980). The role of the lymphatic system in drainage of cerebrospinal fluid and aqueous humour. J. Physiol..

[76] Cserr H.F., Harling-Berg C.J., Knopf P.M. (1992). Drainage of brain extracellular fluid into blood and deep cervical lymph and its immunological significance. Brain Pathol..

[77] Goodman J.R., Adham Z.O., Woltjer R.L., Lund A.W., Iliff J.J. (2018). Characterization of dural sinus-associated lymphatic vasculature in human Alzheimer’s dementia subjects. Brain Behav. Immun..

[78] Kalaria R.N., Ballard C. (1999). Overlap between pathology of Alzheimer disease and vascular dementia. Alzheimer Dis. Assoc. Disord..

[79] Braak H., Braak E. (1995). Staging of Alzheimer’s disease-related neurofibrillary changes. Neurobiol. Aging.

[80] Mirra S.S., Heyman A., McKeel D., Sumi S.M., Crain B.J., Brownlee L.M., Vogel F.S., Hughes J.P., van Belle G., Berg L. (1991). The Consortium to Establish a Registry for Alzheimer’s Disease (CERAD). Part II. Standardization of the neuropathologic assessment of Alzheimer’s disease. Neurology.

[81] Louveau A., Plog B.A., Antila S., Alitalo K., Nedergaard M., Kipnis J. (2017). Understanding the functions and relationships of the glymphatic system and meningeal lymphatics. J. Clin. Investig..

[82] Carare R.O., Bernardes-Silva M., Newman T.A., Page A.M., Nicoll J.A.R., Perry V.H., Weller R.O. (2008). Solutes, but not cells, drain from the brain parenchyma along basement membranes of capillaries and arteries: Significance for cerebral amyloid angiopathy and neuroimmunology. Neuropathol. Appl. Neurobiol..

[83] Kayed R., Head E., Sarsoza F., Saing T., Cotman C.W., Necula M., Margol L., Wu J., Breydo L., Thompson J.L. (2007). Fibril specific, conformation dependent antibodies recognize a generic epitope common to amyloid fibrils and fibrillar oligomers that is absent in prefibrillar oligomers. Mol. Neurodegener..

[84] Aho L., Pikkarainen M., Hiltunen M., Leinonen V., Alafuzoff I. (2010). Immunohistochemical visualization of amyloid-β protein precursor and amyloid-β in extra- and intracellular compartments in the human brain. J. Alzheimer’s Dis..

[85] Liu P., Paulson J.B., Forster C.L., Shapiro S.L., Ashe K.H., Zahs K.R. (2015). Characterization of a novel mouse model of Alzheimer’s disease—Amyloid pathology and unique β-amyloid oligomer profile. PLoS ONE.

[86] Rak M., Del Bigio M.R., Mai S., Westaway D., Gough K. (2007). Dense-core and diffuse Aβ plaques in TgCRND8 mice studied with synchrotron FTIR microspectroscopy. Biopolymers.

[87] Trumbore C.N. (2016). Shear-induced Amyloid formation in the brain: I. Potential vascular and Parenchymal processes. J. Alzheimer’s Dis..

[88] Bedussi B., Naessens D.M.P., de Vos J., Olde Engberink R., Wilhelmus M.M.M., Richard E., ten Hove M., vanBavel E., Bakker E.N.T.P. (2017). Enhanced interstitial fluid drainage in the hippocampus of spontaneously hypertensive rats. Sci. Rep..

[89] Johnston M., Zakharov A., Papaiconomou C., Salmasi G., Armstrong D. (2004). Evidence of connections between cerebrospinal fluid and nasal lymphatic vessels in humans, non-human primates and other mammalian species. Cerebrospinal Fluid. Res..

[90] Song A.K., Hett K., Eisma J.J., McKnight C.D., Elenberger J., Stark A.J., Kang H., Yan Y., Considine C.M., Donahue M.J. (2023). Parasagittal dural space hypertrophy and amyloid-β deposition in Alzheimer’s disease. Brain Commun..

[91] Jagust W.J., Landau S.M., Alzheimer’s Disease Neuroimaging Initiative (2021). Temporal dynamics of β-amyloid accumulation in aging and Alzheimer disease. Neurology.

[92] Ikonomovic M.D., Klunk W.E., Abrahamson E.E., Mathis C.A., Price J.C., Tsopelas N.D., Lopresti B.J., Ziolko S., Bi W., Paljug W.R. (2008). Post-mortem correlates of in vivo PiB-PET amyloid imaging in a typical case of Alzheimer’s disease. Brain.

[93] Klunk W.E., Engler H., Nordberg A., Wang Y., Blomqvist G., Holt D.P., Bergström M., Savitcheva I., Huang G., Estrada S. (2004). Imaging brain amyloid in Alzheimer’s disease with Pittsburgh Compound-B. Ann. Neurol..

[94] Jack C.R., Lowe V.J., Senjem M.L., Weigand S.D., Kemp B.J., Shiung M.M., Knopman D.S., Boeve B.F., Klunk W.E., Mathis C.A. (2008). 11C PiB and structural MRI provide complementary information in imaging of Alzheimer’s disease and amnestic mild cognitive impairment. Brain.

[95] Svedberg M.M., Hall H., Hellström-Lindahl E., Estrada S., Guan Z., Nordberg A., Långström B. (2009). [11C]PIB-amyloid binding and levels of Aβ40 and Aβ42 in postmortem brain tissue from Alzheimer patients. Neurochem. Int..

[96] Cataldo A.M., Nixon R.A. (1990). Enzymatically active lysosomal proteases are associated with amyloid deposits in Alzheimer brain. Proc. Natl. Acad. Sci. USA.

[97] Yang W., Wu Q., Yuan C., Gao J., Xiao M., Gu M., Ding J., Hu G. (2012). Aquaporin-4 mediates astrocyte response to β-amyloid. Mol. Cell. Neurosci..

[98] Bedussi B., van Lier M.G.J.T.B., Bartstra J.W., de Vos J., Siebes M., VanBavel E., Bakker E.N.T.P. (2015). Clearance from the mouse brain by convection of interstitial fluid towards the ventricular system. Fluids Barriers CNS.

[99] de Rougemont J., Ames A., Nesbett F.B., Hofmann H.F. (1960). Fluid formed by choroid plexus: A technique for its collection and a comparison of its electrolyte composition with serum and cisternal fluids. J. Neurophysiol..

[100] Rennels M.L., Gregory T.F., Blaumanis O.R., Fujimoto K., Grady P.A. (1985). Evidence for a ‘paravascular’ fluid circulation in the mammalian central nervous system, provided by the rapid distribution of tracer protein throughout the brain from the subarachnoid space. Brain Res..

[101] Xie L., Kang H., Xu Q., Chen M.J., Liao Y., Thiyagarajan M., O’Donnell J., Christensen D.J., Nicholson C., Iliff J.J. (2013). Sleep Drives Metabolite Clearance from the Adult Brain. Science.

[102] Iliff J.J., Chen M.J., Plog B.A., Zeppenfeld D.M., Soltero M., Yang L., Singh I., Deane R., Nedergaard M. (2014). Impairment of Glymphatic Pathway Function Promotes Tau Pathology after Traumatic Brain Injury. J. Neurosci..

[103] Ringstad G., Eide P.K. (2020). Cerebrospinal fluid tracer efflux to parasagittal dura in humans. Nat. Commun..

[104] Eide P.K., Ringstad G. (2021). Cerebrospinal fluid egress to human parasagittal dura and the impact of sleep deprivation. Brain Res..

[105] Park M., Park J.P., Kim S.H., Cha Y.J. (2022). Evaluation of dural channels in the human parasagittal dural space and dura mater. Ann. Anat..

[106] Hett K., McKnight C.D., Eisma J.J., Elenberger J., Lindsey J.S., Considine C.M., Claassen D.O., Donahue M.J. (2022). Parasagittal dural space and cerebrospinal fluid (CSF) flow across the lifespan in healthy adults. Fluids Barriers CNS.

[107] Eisma J.J., McKnight C.D., Hett K., Elenberger J., Song A.K., Stark A.J., Claassen D.O., Donahue M.J. (2022). Choroid plexus perfusion and bulk cerebrospinal fluid flow across the adult lifespan. J. Cereb. Blood Flow Metab..

[108] Li Y., Rusinek H., Butler T., Glodzik L., Pirraglia E., Babich J., Mozley P.D., Nehmeh S., Pahlajani S., Wang X. (2022). Decreased CSF clearance and increased brain amyloid in Alzheimer’s disease. Fluids Barriers CNS.

[109] de Leon M.J., Li Y., Okamura N., Tsui W.H., Saint-Louis L.A., Glodzik L., Osorio R.S., Fortea J., Butler T., Pirraglia E. (2017). Cerebrospinal Fluid Clearance in Alzheimer Disease Measured with Dynamic PET. J. Nucl. Med..

[110] Park M., Kim J.W., Ahn S.J., Cha Y.J., Suh S.H. (2020). Aging is positively associated with peri-sinus lymphatic space volume: Assessment using 3 T black-blood MRI. J. Clin. Med..

[111] Ismail R., Parbo P., Madsen L.S., Hansen A.K., Hansen K.V., Schaldemose J.L., Kjeldsen P.L., Stokholm M.G., Gottrup H., Eskildsen S.F. (2020). The relationships between neuroinflammation, beta-amyloid and tau deposition in Alzheimer’s disease: A longitudinal PET study. J. Neuroinflam..

[112] Kovacs G.G. (2016). Molecular pathological classification of neurodegenerative diseases: Turning towards precision medicine. Int. J. Mol. Sci..

[113] Brettschneider J., Del Tredici K., Lee V.M., Trojanowski J.Q. (2015). Spreading of pathology in neurodegenerative diseases: A focus on human studies. Nat. Rev. Neurosci..

[114] Eisele Y.S., Duyckaerts C. (2016). Propagation of Aβ pathology: Hypotheses, discoveries, and yet unresolved questions from experimental and human brain studies. Acta Neuropathol..

[115] Guo J.L., Lee V.M. (2014). Cell-to-cell transmission of pathogenic proteins in neurodegenerative diseases. Nat. Med..

[116] Uchihara T., Giasson B.I., Paulus W. (2016). Propagation of Abeta, tau and alpha-synuclein pathology between experimental models and human reality: Prions, propagons and propaganda. Acta Neuropathol..

[117] Frontzek K., Lutz M.I., Aguzzi A., Kovacs G.G., Budka H. (2016). Amyloid-beta pathology and cerebral amyloid angiopathy are frequent in iatrogenic Creutzfeldt-Jakob disease after dural grafting. Swiss Med. Wkly..

[118] Preusser M., Ströbel T., Gelpi E., Eiler M., Broessner G., Schmutzhard E., Budka H. (2006). Alzheimer-type neuropathology in a 28 year old patient with iatrogenic Creutzfeldt-Jakob disease after dural grafting. J. Neurol. Neurosurg. Psychiatry.

[119] Duyckaerts C., Delatour B., Potier M.C. (2009). Classification and basic pathology of Alzheimer disease. Acta Neuropathol..

[120] Metsaars W.P., Hauw J.J., van Welsem M.E., Duyckaerts C. (2003). A grading system of Alzheimer disease lesions in neocortical areas. Neurobiol. Aging.

[121] Thal D.R., Rub U., Orantes M., Braak H. (2002). Phases of A beta deposition in the human brain and its relevance for the development of AD. Neurology.

[122] Cupidi C., Capobianco R., Goffredo D., Marcon G., Ghetti B., Bugiani O., Tagliavini F., Giaccone G. (2010). Neocortical variation of Abeta load in fully expressed pure Alzheimer’s disease. J. Alzheimers Dis..

[123] Thal D.R., Ghebremedhin E., Rub U., Yamaguchi H., Del Tredici K., Braak H. (2002). Two types of sporadic cerebral amyloid angiopathy. J. Neuropathol. Exp. Neurol..

[124] Thal D.R., Griffin W.S., de Vos R.A., Ghebremedhin E. (2008). Cerebral amyloid angiopathy and its relationship to Alzheimer’s disease. Acta Neuropathol..

[125] Pletnikova O., Rudow G.L., Hyde T.M., Kleinman J.E., Ali S.Z.B., Bharadwaj R., Gangadeen S., Crain B.J., Fowler D.R.M., Rubio A.I. (2015). Alzheimer lesions in the autopsied brains of people 30 to 50 years of age. Cogn. Behav. Neurol..

[126] Braak H., Del Tredici K. (2011). The pathological process underlying Alzheimer’s disease in individuals under thirty. Acta Neuropathol..

[127] Braak H., Del Tredici K. (2015). The preclinical phase of the pathological process underlying sporadic Alzheimer’s disease. Brain.

[128] Grinberg L.T., Rüb U., Ferretti R.E.L., Nitrini R., Farfel J.M., Polichiso L., Gierga K., Jacob-Filho W., Heinsen H., Brazilian Brain Bank Study Group (2009). The dorsal raphe nucleus shows phospho-tau neurofibrillary changes before the transentorhinal region in Alzheimer’s disease. A precocious onset?. Neuropathol. Appl. Neurobiol..

[129] Stratmann K., Heinsen H., Korf H., Del Turco D., Ghebremedhin E., Seidel K., Bouzrou M., Grinberg L.T., Bohl J., Wharton S.B. (2015). Precortical Phase of Alzheimer’s Disease (AD)-Related Tau Cytoskeletal Pathology. Brain Pathol..

[130] Johnson V.E., Stewart W., Smith D.H. (2010). Traumatic brain injury and amyloid-beta pathology: A link to Alzheimer’s disease?. Nat. Rev. Neurosci..

[131] Johnson V.E., Stewart W., Smith D.H. (2012). Widespread tau and amyloid-beta pathology many years after a single traumatic brain injury in humans. Brain Pathol..

[132] McKee A.C., Stein T.D., Kiernan P.T., Alvarez V.E. (2015). The neuropathology of chronic traumatic encephalopathy. Brain Pathol..

[133] McKee A.C., Stern R.A., Nowinski C.J., Stern R.A., Daneshvar D.H., Alvarez V.E., Lee H.-S., Hall G., Wojtowicz S.M., Baugh C.M. (2013). The spectrum of disease in chronic traumatic encephalopathy. Brain.

[134] Zedde M., Piazza F., Pascarella R. (2025). Traumatic Brain Injury and Chronic Traumatic Encephalopathy: Not Only Trigger for Neurodegeneration but Also for Cerebral Amyloid Angiopathy?. Biomedicines.

[135] McKee A.C., Cairns N.J., Dickson D.W., Folkerth R.D., Keene C.D., Litvan I., Perl D.P., Stein T.D., Vonsattel J.-P. (2016). The first NINDS/NIBIB consensus meeting to define neuropathological criteria for the diagnosis of chronic traumatic encephalopathy. Acta Neuropathol..

[136] Kovacs G.G., Lutz M.I., Ricken G., Ströbel T., Höftberger R., Preusser M., Regelsberger G., Hönigschnabl S., Reiner A., Fischer P. (2016). Dura mater is a potential source of Aβ seeds. Acta Neuropathol..

[137] Kovacs G.G., Milenkovic I., Wöhrer A., Höftberger R., Gelpi E., Haberler C., Hönigschnabl S., Reiner-Concin A., Heinzl H., Jungwirth S. (2013). Non-Alzheimer neurodegenerative pathologies and their combinations are more frequent than commonly believed in the elderly brain: A community-based autopsy series. Acta Neuropathol..

[138] Parchi P., de Boni L., Saverioni D., Cohen M.L., Ferrer I., Gambetti P., Gelpi E., Giaccone G., Hauw J.-J., Höftberger R. (2012). Consensus classification of human prion disease histotypes allows reliable identification of molecular subtypes: An inter-rater study among surveillance centres in Europe and USA. Acta Neuropathol..

[139] Alafuzoff I., Thal D.R., Arzberger T., Bogdanovic N., Al-Sarraj S., Bodi I., Boluda S., Bugiani O., Duyckaerts C., Gelpi E. (2009). Assessment of β-amyloid deposits in human brain: A study of the BrainNet Europe Consortium. Acta Neuropathol..

[140] Delaere P., Duyckaerts C., He Y., Piette F., Hauw J.J. (1991). Subtypes and differential laminar distributions of beta A4 deposits in Alzheimer’s disease: Relationship with the intellectual status of 26 cases. Acta Neuropathol..

[141] Ghoshal N., Cali I., Perrin R.J., Josephson S.A., Sun N., Gambetti P., Morris J.C. (2009). Codistribution of Amyloid β Plaques and Spongiform Degeneration in Familial Creutzfeldt-Jakob Disease With the E200K-129M Haplotype. Arch. Neurol..

[142] Kovacs G.G., Seguin J., Quadrio I., Höftberger R., Kapás I., Streichenberger N., Biacabe A.G., Meyronet D., Sciot R., Vandenberghe R. (2011). Genetic Creutzfeldt-Jakob disease associated with the E200K mutation: Characterization of a complex proteinopathy. Acta Neuropathol..

[143] Reiniger L., Lukic A., Linehan J., Rudge P., Collinge J., Mead S., Brandner S. (2011). Tau, prions and Aβ: The triad of neurodegeneration. Acta Neuropathol..

[144] Kovacs G.G., Horvath M.C., Majtenyi K., Lutz M.I., Hurd Y.L., Keller E. (2015). Heroin abuse exaggerates age-related deposition of hyperphosphorylated tau and p62-positive inclusions. Neurobiol. Aging.

[145] Chen X.H., Johnson V.E., Uryu K., Trojanowski J.Q., Smith D.H. (2009). A lack of amyloid beta plaques despite persistent accumulation of amyloid beta in axons of long-term survivors of traumatic brain injury. Brain Pathol..

[146] Horsburgh K., Cole G.M., Yang F. (2000). beta-amyloid (Abeta)42(43), abeta42, abeta40 and apoE immunostaining of plaques in fatal head injury. Neuropathol. Appl. Neurobiol..

[147] Scott G., Ramlackhansingh A.F., Edison P., Hellyer P., Cole J., Veronese M., Leech R., Greenwood R.J., Turkheimer F.E., Gentleman S.M. (2016). Amyloid pathology and axonal injury after brain trauma. Neurology.

[148] Tokuda T., Ikeda S., Yanagisawa N., Ihara Y., Glenner G.G. (1991). Re-examination of ex-boxers’ brains using immunohistochemistry with antibodies to amyloid beta-protein and tau protein. Acta Neuropathol..

[149] Irwin D.J., Abrams J.Y., Schonberger L.B., Leschek E.W., Mills J.L., Lee V.M.-Y., Trojanowski J.Q. (2013). Evaluation of potential infectivity of Alzheimer and Parkinson disease proteins in recipients of cadaver-derived human growth hormone. JAMA Neurol..

[150] Bohl J., Storkel S., Steinmetz H. (1989). Involvement of the central nervous system and its coverings in different forms of amyloidosis. Prog. Clin. Biol. Res..

[151] Andres K.H. (1967). On the fine structure of the arachnoidea and dura mater of mammals. Z. Zellforsch. Mikrosk. Anat..

[152] Roland J., Bernard C., Bracard S., Czorny A., Floquet J., Race J.M., Forlodou P., Picard L. (1987). Microvascularization of the intracranial dura mater. Surg. Radiol. Anat..

[153] Attems J., Jellinger K., Thal D.R., Van Nostrand W. (2011). Review: Sporadic cerebral amyloid angiopathy. Neuropathol. Appl. Neurobiol..

[154] Carare R.O., Hawkes C.A., Jeffrey M., Kalaria R.N., Weller R.O. (2013). Review: Cerebral amyloid angiopathy, prion angiopathy, CADASIL and the spectrum of protein elimination failure angiopathies (PEFA) in neurodegenerative disease with a focus on therapy. Neuropathol. Appl. Neurobiol..

[155] Weller R.O., Subash M., Preston S.D., Mazanti I., Carare R.O. (2008). Perivascular drainage of amyloid-beta peptides from the brain and its failure in cerebral amyloid angiopathy and Alzheimer’s disease. Brain Pathol..

[156] Xia L., Min W., Lu X., Wang C., Jiang Z., Zhang Y., Ye S., Su Z., Zheng W., Liu H. (2017). Subdural Hemorrhage from Cerebral Amyloid Angiopathy–Related Intracerebral Hemorrhage: A Risk Factor for Postoperative Hemorrhage. World Neurosurg..

[157] Zupan M., Straus L., Velnar T., Bošnjak M., Jensen-Kondering U., Splavski B., Frol S. (2025). Coexisting Subdural Hematoma in Cerebral Amyloid Angiopathy: A Case Series. Neurol. Int..

[158] Bruce S.S., Murthy S.B., Parikh N.S., Schweitzer A.D., Roytman M., Liechty B., Cisse B., Kirou K.A., Navi B.B. (2024). Cerebral Amyloid Angiopathy-Related Inflammation Presenting as Nontraumatic Subdural Hematoma. Ann. Intern. Med. Clin. Cases.

[159] Andres W., Bruce S., Merkler A.E., Iadecola C., De Leon M.J., Chiang G.C., Kamel H., Zhang C., Murthy S.B. (2025). Association Between Cerebral Amyloid Angiopathy and Nontraumatic Subdural Hemorrhage. Neurology.

[160] Rivier C.A., Kamel H., Sheth K.N., Iadecola C., Gupta A., de Leon M.J., Ross E., Falcone G.J., Murthy S.B. (2023). Cerebral Amyloid Angiopathy and Risk of Isolated Nontraumatic Subdural Hemorrhage. JAMA Neurol..

[161] Keage H.A., Carare R.O., Friedland R.P., Ince P.G., Love S., Nicoll J.A., Wharton S.B., Weller R.O., Brayne C. (2009). Population studies of sporadic cerebral amyloid angiopathy and dementia: A systematic review. BMC Neurol..

[162] De Kort A.M., Verbeek M.M., Schreuder F.H.B.M., Klijn C.J.M., Jäkel L. (2024). Prevalence of Cerebral Amyloid Angiopathy Pathology and Strictly Lobar Microbleeds in East-Asian Versus Western Populations: A Systematic Review and Meta-Analysis. J. Stroke.

[163] Yamada M., Naiki H. (2012). Cerebral amyloid angiopathy. Prog. Mol. Biol. Transl. Sci..

[164] Husain M.A., Laurent B., Plourde M. (2021). APOE and Alzheimer’s Disease: From Lipid Transport to Physiopathology and Therapeutics. Front. Neurosci..

[165] Nicoll J.A., Burnett C., Love S., Graham D.I., Dewar D., Ironside J.W., Stewart J., Vinters H.V. (1997). High frequency of apolipoprotein E epsilon 2 allele in hemorrhage due to cerebral amyloid angiopathy. Ann. Neurol..

[166] Li Z., Shue F., Zhao N., Shinohara M., Bu G. (2020). APOE2: Protective mechanism and therapeutic implications for Alzheimer’s disease. Mol. Neurodegener..

[167] Yu L., Boyle P.A., Nag S., Leurgans S., Buchman A.S., Wilson R.S., Arvanitakis Z., Farfel J.M., De Jager P.L., Bennett D.A. (2015). APOE and cerebral amyloid angiopathy in community-dwelling older persons. Neurobiol. Aging.

[168] Mann D.M.A., Davidson Y.S., Robinson A.C., Allen N., Hashimoto T., Richardson A., Jones M., Snowden J.S., Pendleton N., Potier M.C. (2018). Patterns and severity of vascular amyloid in Alzheimer’s disease associated with duplications and missense mutations in APP gene, Down syndrome and sporadic Alzheimer’s disease. Acta Neuropathol..

[169] Bellenguez C., Küçükali F., Jansen I.E., Kleineidam L., Moreno-Grau S., Amin N., Naj A.C., Campos-Martin R., Grenier-Boley B., Andrade V. (2022). New insights into the genetic etiology of Alzheimer’s disease and related dementias. Nat. Genet..

[170] Greenberg S.M., Vonsattel J.P., Stakes J.W., Gruber M., Finklestein S.P. (1993). The clinical spectrum of cerebral amyloid angiopathy: Presentations without lobar hemorrhage. Neurology.

[171] Vonsattel J.P., Myers R.H., Hedley-Whyte E.T., Ropper A.H., Bird E.D., Richardson E.P. (1991). Cerebral amyloid angiopathy without and with cerebral hemorrhages: A comparative histological study. Ann. Neurol..

[172] Vinters H.V. (1987). Cerebral amyloid angiopathy. A critical review. Stroke.

[173] Vinters H.V., Natte R., Maat S.M., van Duinen S.G., Hegeman K., Welling G.C., Haan J., Roos R.A. (1998). Secondary microvascular degeneration in amyloid angiopathy of patients with hereditary cerebral hemorrhage with amyloidosis, Dutch type (HCHWA-D). Acta Neuropathol..

[174] Thomas T., McLendon C., Sutton E.T., Thomas G. (1997). Beta-amyloid-induced cerebrovascular endothelial dysfunction. Ann. N. Y. Acad. Sci..

[175] Van Nostrand W.E., Davis-Salinas J., SaporitoIrwin S.M. (1996). Amyloid beta-protein induces the cerebrovascular cellular pathology of Alzheimer’s disease related disorders. Ann. N. Y. Acad. Sci..

[176] Davis-Salinas J., Saporito-Irwin S.M., Van Nostrand W.E. (1995). Amyloid beta-protein induces its own production in cultured degenerating cerebrovascular smooth muscle cells. J. Neurochem..

[177] Verbeek M.M., de Waal R.M., Schipper J.J., Van Nostrand W.E. (1997). Rapid degeneration of cultured human brain pericytes by amyloid beta protein. J. Neurochem..

[178] Roher A.E., Lowenson J.D., Clarke S., Woods A.S., Cotter R.J., Gowing E., Ball M.J. (1993). Beta-amyloid-(1–42) is a major component of cerebrovascular amyloid deposits: Implications for the pathology of Alzheimer disease. Proc. Natl. Acad. Sci. USA.

[179] van Horssen J., de Jong D., de Waal R.M., Maass C., Otte-Holler I., Kremer B., Verbeek M.M., Wesseling P. (2005). Cerebral amyloid angiopathy with severe secondary vascular pathology: A histopathological study. Dement. Geriatr. Cogn. Disord..

[180] Zedde M., Piazza F., Pascarella R. (2025). Clinical and Neuroradiological Manifestations of Cerebral Amyloid Angiopathy: A Closer Look into the Natural History of a Frequent Disease. J. Clin. Med..

[181] Zedde M., Pascarella R., Piazza F. (2023). CAA-ri and ARIA: Two Faces of the Same Coin?. AJNR Am. J. Neuroradiol..

[182] Piazza F., Basso G., Pascarella R., Carare R.O., Zedde M. (2022). Author Response: Spontaneous ARIA-like Events in Cerebral Amyloid Angiopathy-Related Inflammation: A Multicenter Prospective Longitudinal Cohort Study. Neurology.

[183] Piazza F., Caminiti S.P., Zedde M., Presotto L., DiFrancesco J.C., Pascarella R., Giossi A., Sessa M., Poli L., Basso G. (2022). Association of Microglial Activation with Spontaneous ARIA-E and CSF Levels of Anti-Aβ Autoantibodies. Neurology.

[184] Antolini L., DiFrancesco J.C., Zedde M., Basso G., Arighi A., Shima A., Cagnin A., Caulo M., Carare R.O., Charidimou A. (2021). Spontaneous ARIA-like Events in Cerebral Amyloid Angiopathy-Related Inflammation: A Multicenter Prospective Longitudinal Cohort Study. Neurology.

[185] Shim G.H., Lau E.C.Y., Huynh A.L.H., Lu C.Y., Tan E.C.K. (2025). Influence of patient characteristics on efficacy and safety of anti-amyloid monoclonal antibodies in Alzheimer’s disease: A systematic review and meta-analysis. Ageing Res. Rev..

[186] Qiao Y., Gu J., Yu M., Chi Y., Ma Y. (2024). Comparative Efficacy and Safety of Monoclonal Antibodies for Cognitive Decline in Patients with Alzheimer’s Disease: A Systematic Review and Network Meta-Analysis. CNS Drugs.

[187] Liu S., Zhao M., Liu Y., Yang X., Yan H., Xu H., Wu Y., Xu Y. (2025). Comparative efficacy and safety of symptomatic therapy and disease-modifying therapy for Alzheimer’s disease: A systematic review and network meta-analysis. Front. Neurosci..

